# Supplementary material for: Spatial Fold Change of FGF Signaling Encodes Positional Information for Segmental Determination in Zebrafish
Source: Cell Rep. Author manuscript; Available in PMC 2018 Jul 27. (PMC6063364; doi:10.1016/j.celrep.2018.06.023)
Supplement: 1 [file NIHMS981694-supplement-1.pdf]

Cell Reports, Volume *24*

Supplemental Information

Spatial Fold Change of FGF Signaling Encodes Positional Information for Segmental Determination in Zebrafish

M. Fethullah Simsek and Ertuğrul M. Özbudak

SUPPLEMENTARY FIGURES AND TABLE

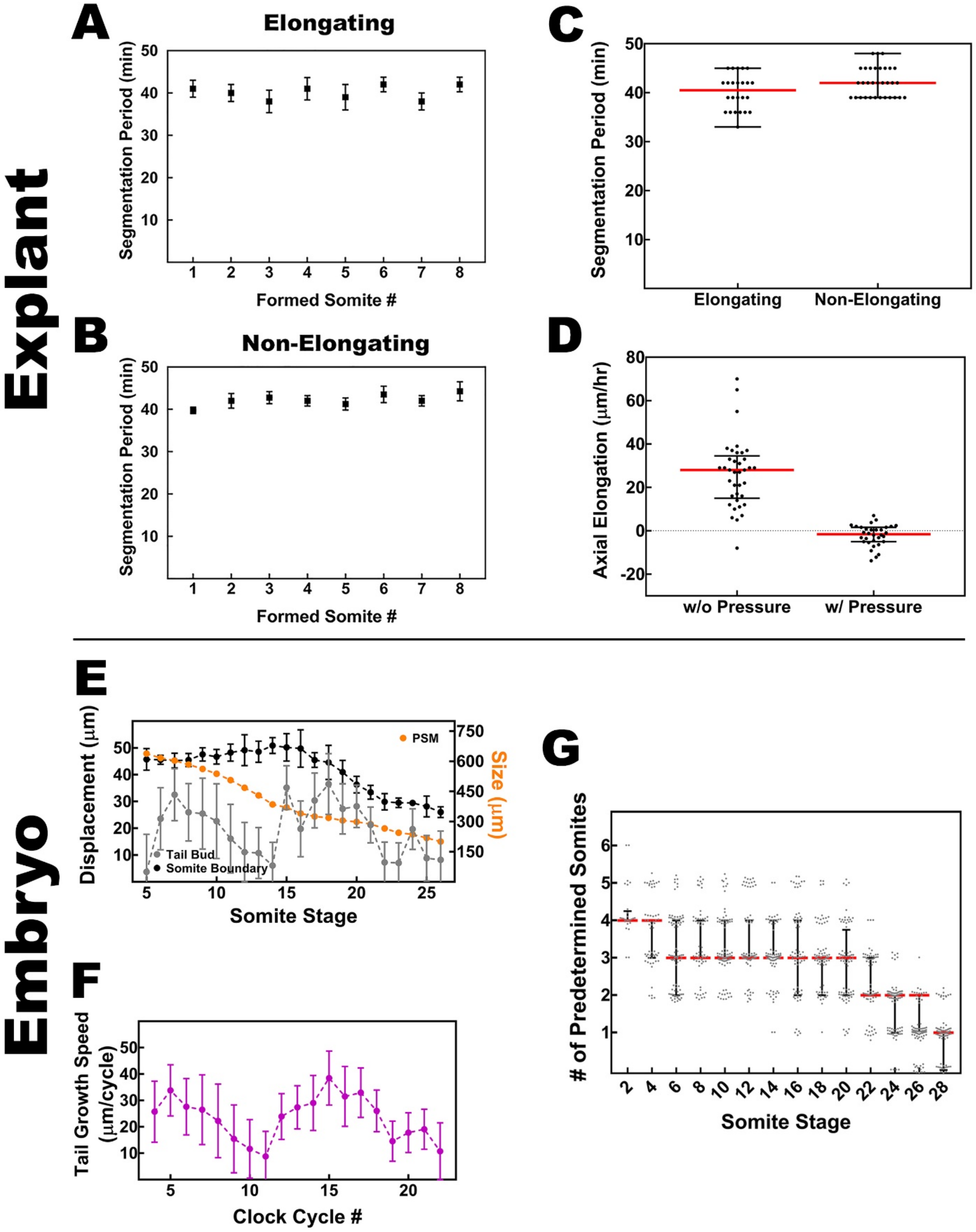

Figure S1. Characterization of 3-D PSM Explants and Quantification of Somitogenesis Dynamics in Intact Embryos, Related to Figures 1, 2, 4, 6, and 8

(A,B) Segmentation period does not change in PSM tissue culture during formation of consecutive somites under elongating (N=4) or non-elongating (N=5) conditions. Error bars indicate s.e.m. (C) Average period also doesn't change in between elongating and non-elongating conditions. Red lines show medians of data. (D) Average axial elongation speeds of explants measured over six hours in culture. Explants stops their axial elongation under pressure (error bars s.d.). (E) Size of presomitic mesoderm (orange, right axis) measured through mid-somitogenesis, from 5 somite stage until 25 somite stage. Displacement of tail bud (gray) in between formation of each somite is calculated from the sum of the change in PSM size and displacement of last somite boundary (size of last formed somite, black). (N=6-20, mean with s.d.). (F) Average axial elongation speed per clock cycle (purple, with s.d.) is calculated from resampling of tail bud displacement from somite stage into segmentation clock cycles using change of predetermined somites data (G). (G) Number of predetermined somites measured, from 2 somite stage until 30 somite stage (N>27). Median values are shown with red lines. Error bars represent quartiles of the data. Original data (integers) are jittered with 0.05 standard deviation keeping mean matching data within 0.01 range, to make data visible (gray dots).

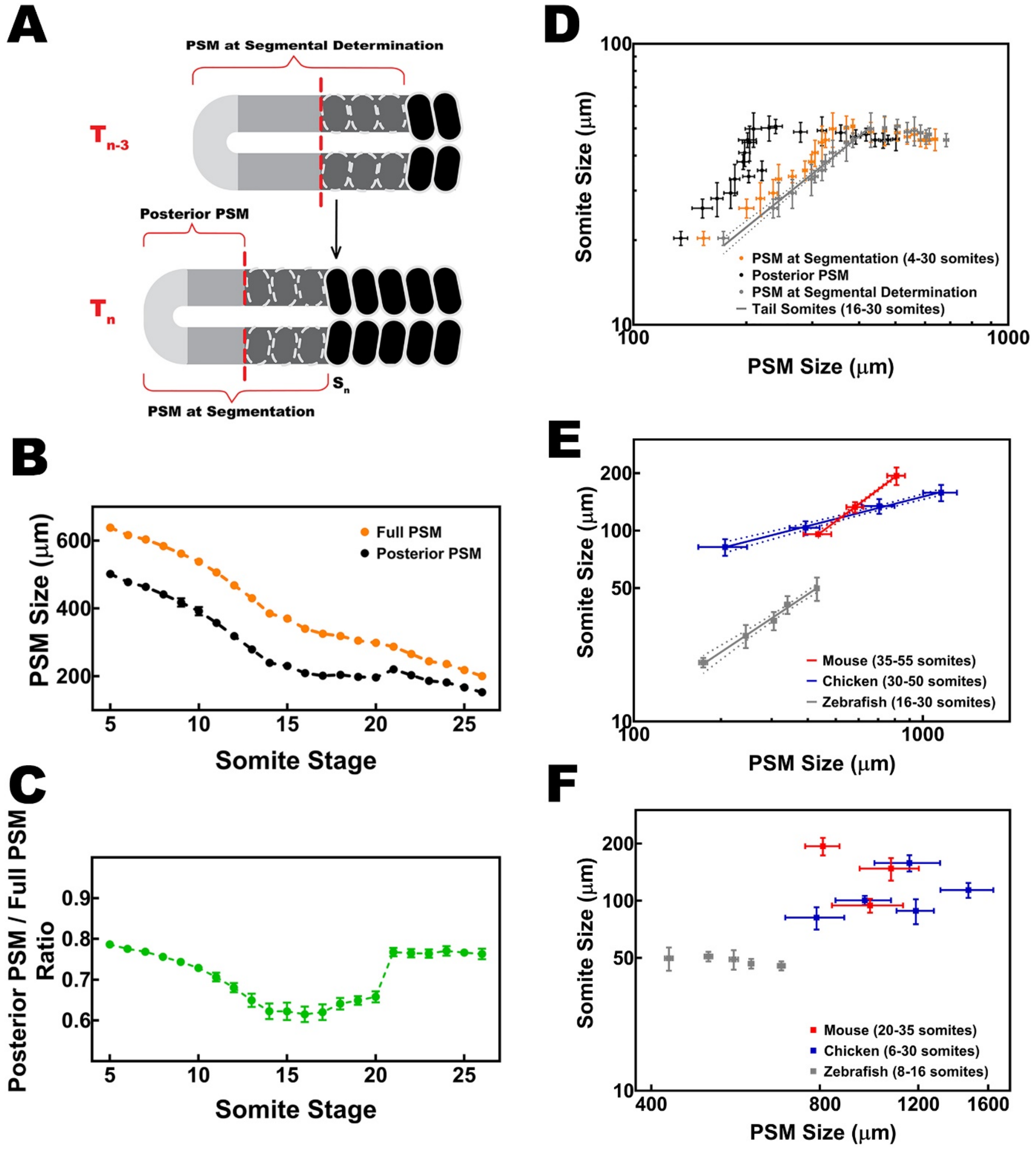

Figure S2. Scaling between Sizes of Tail Somites and the PSM Tissue, Related to Figures 1, and 4

(A) Sketch of PSM and last formed somites at a stage, S_n, when there are three predetermined somites in the anterior PSM (bottom) and the PSM and last formed somites when that n^{th} somite was determined (top). (B) Size measurements for these tissues through somitogenesis. (C) The ratio of posterior PSM versus full PSM is not kept constant. The relative position of the determination front nonlinearly changes in the PSM. (D) The sizes of tail somites scales only with the size of PSM at segmental determination but not with the size of PSM at segmentation or posterior PSM. The anterior somites do not display scaling. (E) Tail somites are segmented in gradually decreasing sizes scaling in size with the PSM at segmental determination for three classes of vertebrate species (reanalyzed from Gomez et al. 2007). (F) The anterior somites do not display scaling in three species. Error bars indicate s.d.

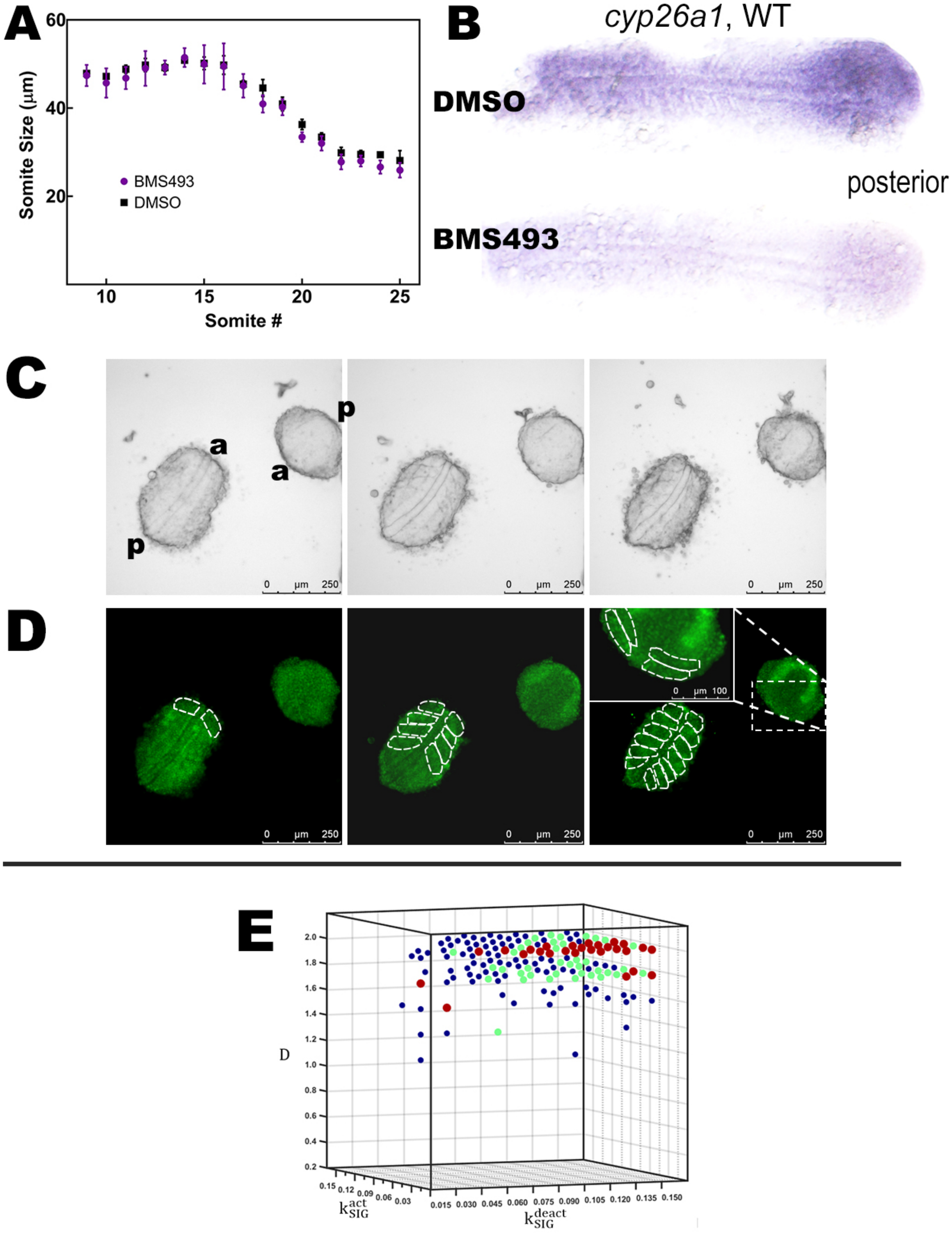

Figure S3. RA Gradient is Inhibited with BMS493 Treatment and Half PSM Explants Wait for Anterior Somites before Segmentation, and Simulations Parameter Space **Related to Figures 2, 3, and 6**

(A) Whole embryos continuously treated with pan-RAR inhibitor BMS493 drug (50 µM for 7 hours, starting at 10 somite stage, N=7) or with DMSO only (1%, corresponding concentration, N=15) formed somite segments with similar sizes. Error bars s.e.m. (B) Transcription of *cyp26a1* is reduced in embryos treated with same concentration of BMS493 for 1 hour compared to DMSO treated embryos. (C) Bright field images of anterior and posterior pieces of the PSM explant without any somites at t=0 (left), t=2 hours (middle) and t=5 hours (right). Short posterior piece of the PSM explant is placed away from and behind the anterior end of the anterior piece and flipped in opposite anteroposterior orientation, to ascertain segmentation is not altered by any kind of morphogen diffusion in between tissues. (D) Images of same explants in green channel showing signal from nuclear expressed GFP marker. Starting from S0 at t=0, borders of forming somites are outlined as dashed white lines. (E) Spread of 243 parameter sets (out of 10,000 tested) giving successful (passing statistical criteria as described in Methods) fit for normal scaling data from PSM explants under non-elongating conditions. X, Y, and Z axes are signal protein deactivation (k_{SIG}^{deact}) and activation (k_{SIG}^{act}) rates and ligand diffusion speed (D) respectively. Different ligand receptor binding rates (k_{LIG}^{bin}), are shown in different colors, size of markers increase as the binding gets stronger. Only three lowest out of ten binding rates gave successful results within the parameter space.

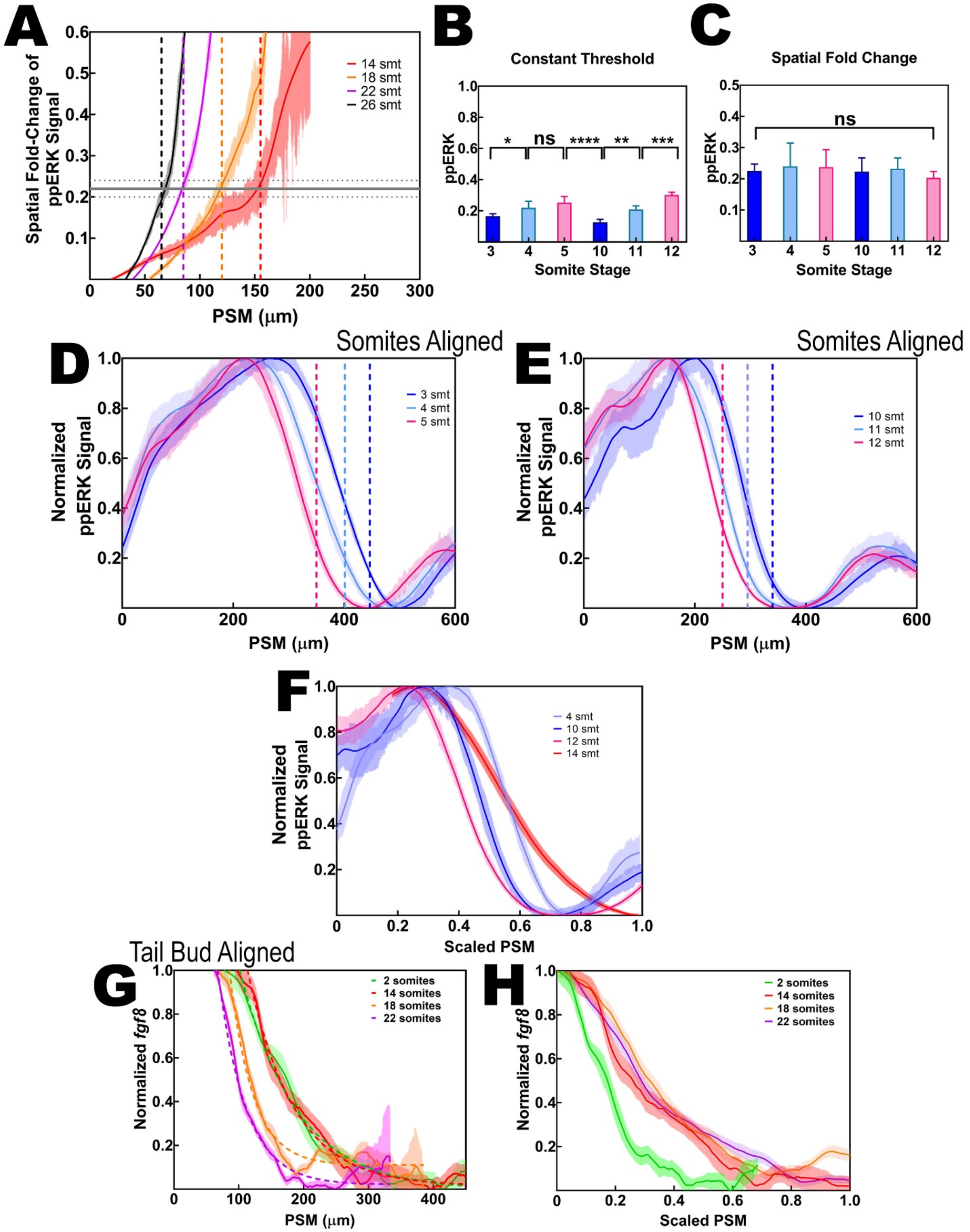

Figure S4. Spatial Fold Change of FGF Signaling at Different Stages and Both *fgf8* mRNA and ppERK Gradients Scale with the PSM size at Late but not Early Stages, Related to Figures 4, and 5

(A) The SFC readout of ppERK levels (n = 17, 9, 14 and 13 for 14, 18, 22 and 26 somite stages, respectively). Posterior end of the PSM are matched for all stages. Determined-undetermined PSM borders are marked by dashed vertical lines with stage matching colors. At the determination front (vertical dashed lines), the SFC of ppERK reads out precisely the same level (22 ± 2%, gray horizontal line) at different stage embryos. (B, C) The SFC (C) but not absolute level (B) of ppERK is conserved at the determination front at 3, 4, 5, 10, 11 and 12 somite staged embryos. Error bars indicate s.d. Note that the absolute level of ppERK is only conserved at stages 4 and 5 somite stages due to the fact that PSM size do not change appreciably at those stages (Gomez et al., 2008), which led to the wrong conclusion that a constant level of ppERK moves stepwise during segmentation (Akiyama et al., 2014). (D, E) Normalized levels of ppERK (n = 20, 14, 16, 14, 12, and 14 for 3, 4, 5, 10, 11, and 12 somite stages) quantified as FGF signaling output. Data is aligned from anterior (formed somites) within each group of consecutive stages. Determined-undetermined PSM borders are marked by dashed vertical lines with stage matching colors, respectively. ppERK profile shows stepwise regression in between consecutive stages determining similar sized somites. (F) Unlike later stages (Figure 4D), ppERK gradient at early stages do not scale with the size of the tissue between posterior tip of notochord and anterior end of the PSM. (G) Normalized levels of *fgf8* mRNA gradient (n = 14, 19, 18, and 18 for 2, 14, 18, and 22 somite stages) quantified as FGF signaling input. (H) *fgf8* mRNA gradient scale with the size of PSM at 14, 18 and 22 somite stages but not at 2 somite stage. Shaded regions are s.e.m. Posterior is left.

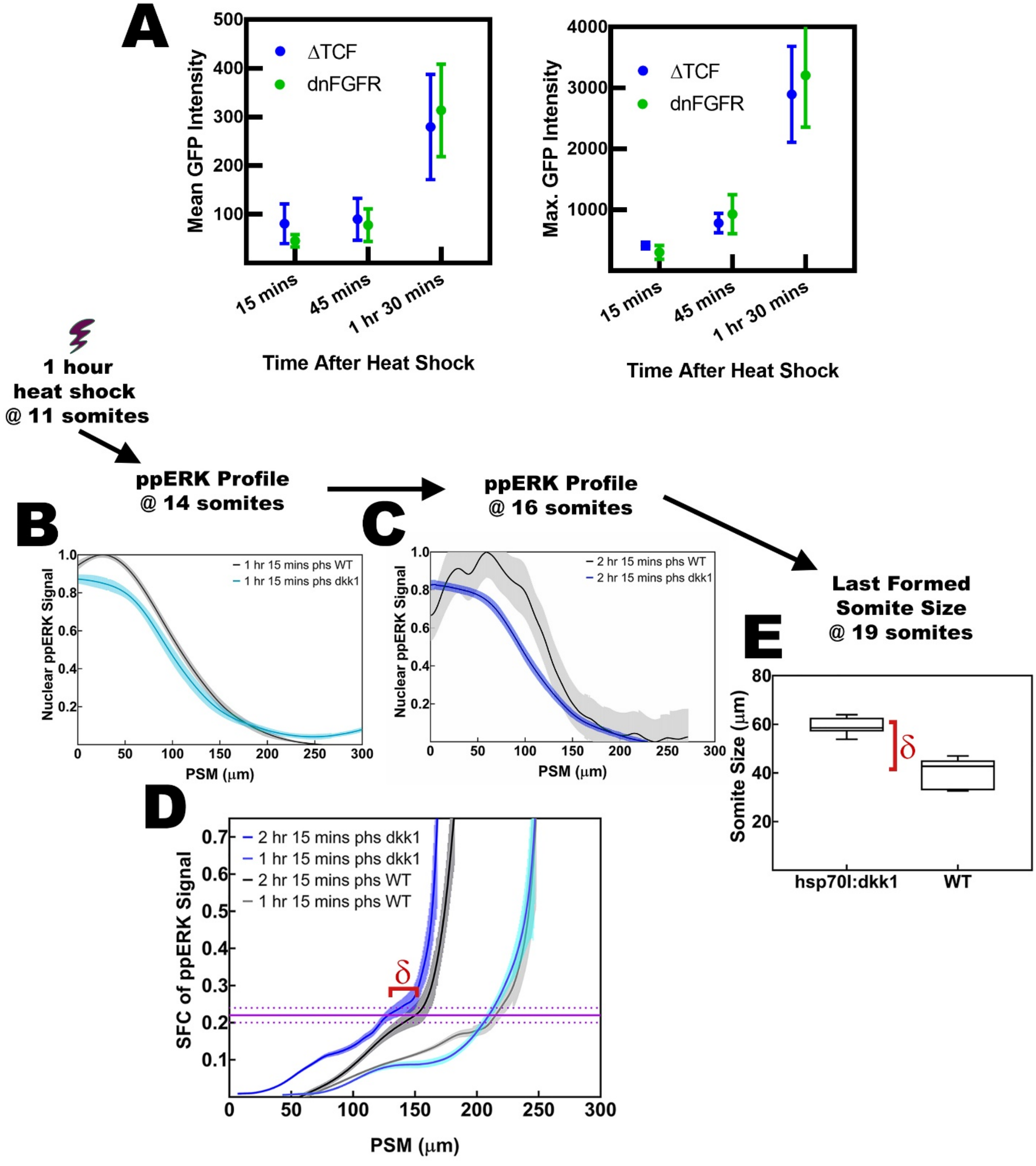

Figure S5. Wnt Signaling Influences Segmental Determination by Acting on the SFC of FGF Signaling, Related to Figure 5

(A) Mean and Maximum GFP intensity accumulated similarly in both *hsp70l:tcf7l1a-GFP* and *hsp70l: dnfgfr1a-EGFP* lines (n=3 and 3, respectively). Error bars indicate s.e.m. (B, C) Perturbation of Wnt signaling affect FGF SFC. Levels of ppERK (green) at 14 (B, n=28) or 16 (C, n=35) somite stages following 1 hour heat-shock (starting at 11 somite stage) in the *hsp70l:dkk1b-GFP* relative to control data (black) for same stages, ([B, n=14] and [C, n=10]). (C) Change of somite sizes following the heat-shock inhibition of Wnt signaling between 12 and 14 somite stages. (D) The SFC of FGF signaling shifted more posteriorly (δ=16+/-2 μm, dark blue) as compared to wild type (black) by 2.25 hours post heat-shock (inhibition of Wnt signaling), but not earlier (light colors), determining size of the first large somite to be formed three cycles later. (B-D) Shaded regions indicate s.e.m. Posterior is left. (E) The increase of somite size, at 19 somite stage, due to Wnt inhibition as quantified from nuclear staining pictures δ=17+/-3 μm (n=15) matches the shift of SFC of ppERK at 16 somite stage (D). Box plots show quartiles of data.

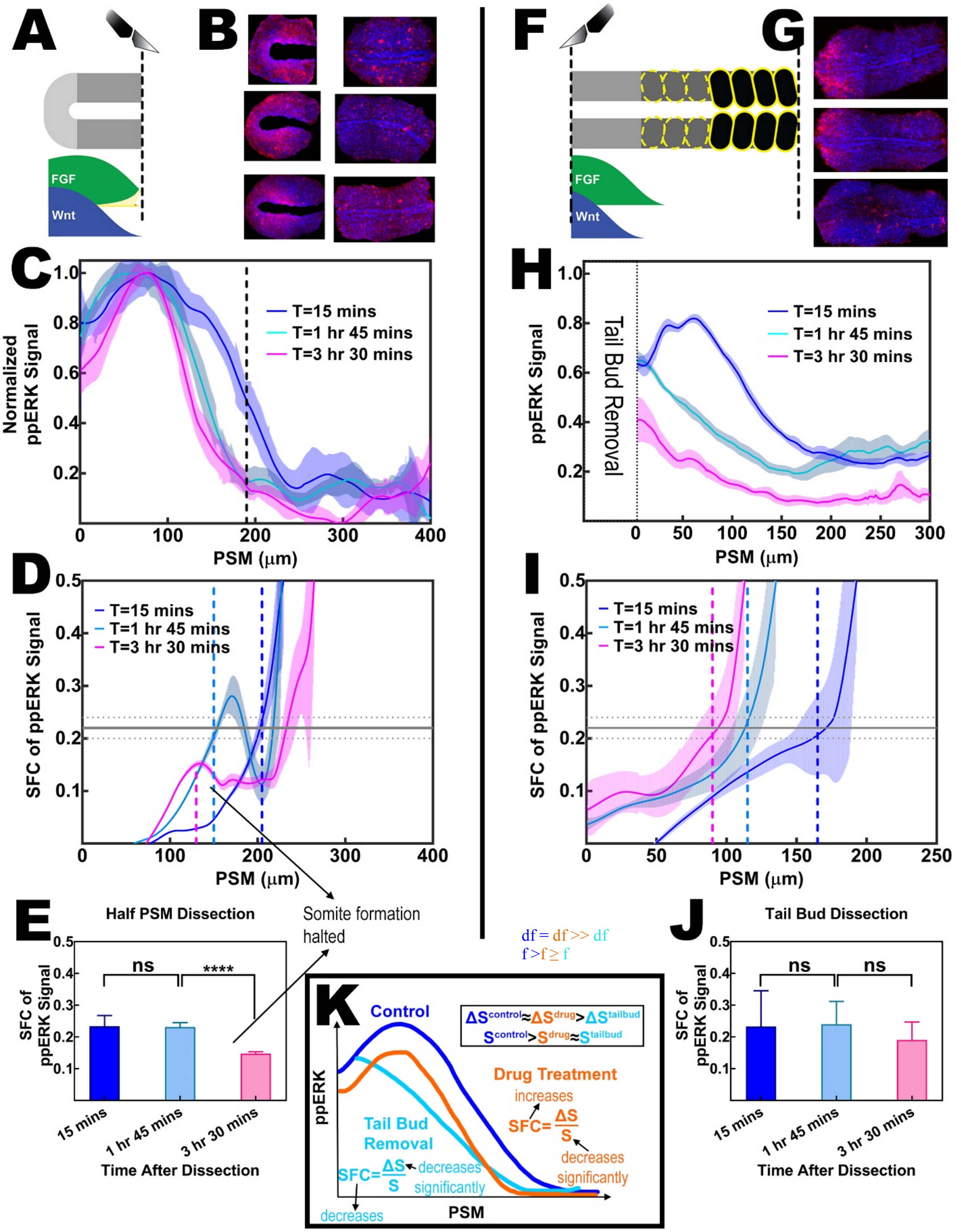

Figure S6. Spatial Fold Change of FGF Signaling Encodes Positional Information for Different Dissections of Explants, Related to Figures 2, and 6

(A, B) ppERK stainings (B) of half PSM explants and their matching anterior PSM halves. (C, D) Normalized levels (C) and the SFC (D) of ppERK quantified (n = 8, 8, and 8 at 15 min, 1.75 hours,and 3.5 hours post surgery). (A, C) Vertical dashed line is the surgery position. (D) Vertical dashed lines are the determination front positions as anticipated from determined somite sizes. (E) The SFC level of ppERK is conserved at the determination front position at 15 min, and 1.75 hours post-surgery when segments successfully formed. Strikingly, in spite of the fact that the PSM is not totally depleted and ppERK activity still establish a gradient, segmentation halts as the SFC of ppERK fails to pass a critical level at 3.5 hours post surgery. (F, G) ppERK stainings (G) of tailbud-truncated explants (F). (H, I) Measured levels (F) and the SFC (G) of ppERK activity (n = 6, 8, and 8 at 15 min, 1.75 hours, and 3.5 hours post surgery). (I) The SFC level of ppERK is conserved at the determination front position at 15 min, 1.75 hours, and 3.5 hours post surgery. Vertical dashed lines are the determination front positions as anticipated from determined somite sizes. Posterior end of the PSM are matched for all stages. Shaded regions and error bars indicate s.e.m. (K) Two different experiments decreasing FGF signaling result opposite effects on somite sizes. Tail bud removal decreases somite sizes whereas drug inhibition of FGF receptor increases. For a specific PSM position, SFC of FGF signaling increases in drug treatment whereas decreases in tail bud removed explants. Posterior is left.

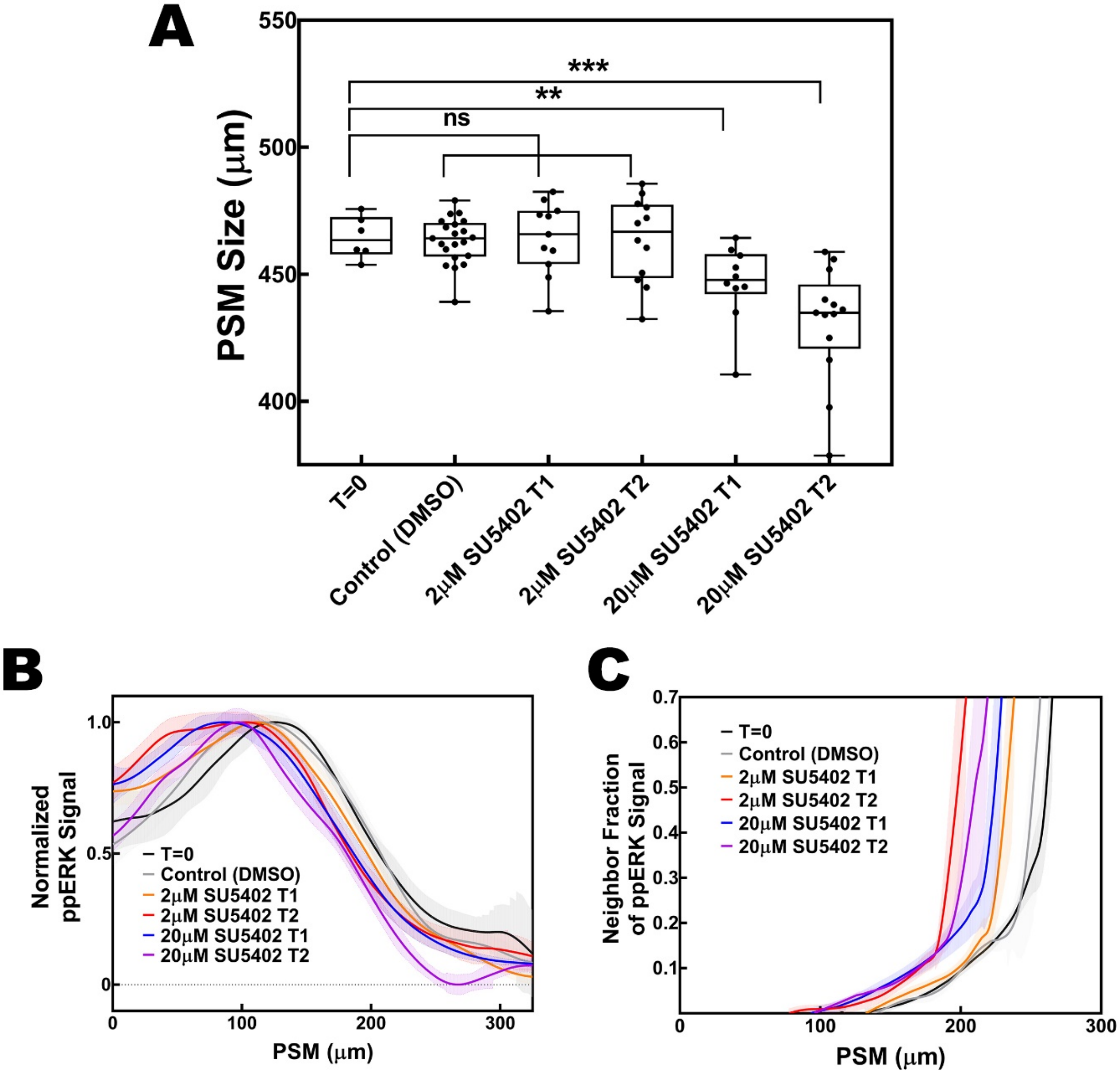

Figure S7. Pharmacological Blocking of FGF Signaling Acts in a Dose Dependent Manner, Related to Figure 6

Embryos were treated with either low (2 μM) or high (20 μM) dose SU5402, or with corresponding DMSO concentrations as control, continuously over 1 or 2 segmentation cycles (T1 and T2). Embryos at 12 somite stage, right after the treatments, were fixed for immunostaining. (A) Sizes of the PSM following low dose SU5402 (n=11) or DMSO (n=21) treatments did not differ from the untreated embryos fixed at same somite stage (T=0, n=6). High dose SU5402 (n=12) treatment resulted in gradually shorter PSM tissue (p=0.0085 and 0.0002, non-parametric student t-test) indicating reduction of axial elongation speed. (B) Changes in the FGF signaling are quantified with immunostaining of normalized nuclear ppERK levels. Inhibition of FGF signaling resulted posterior shift of normalized gradients. High dose inhibition resulted posterior shift immediately within the first segmentation cycle whereas low dose SU5402 caught the shift in two cycles. (C) The SFC of FGF signaling shifted gradually over two cycles for low dose treatment determining bigger somite in the second cycle of the treatment. The shift for the high dose treatment was similar for both short and high duration treatments showing somite sizes reduce back to normal after a single large somite, due to reduction of axial elongation speed. Shaded regions indicate s.e.m. Posterior is left.

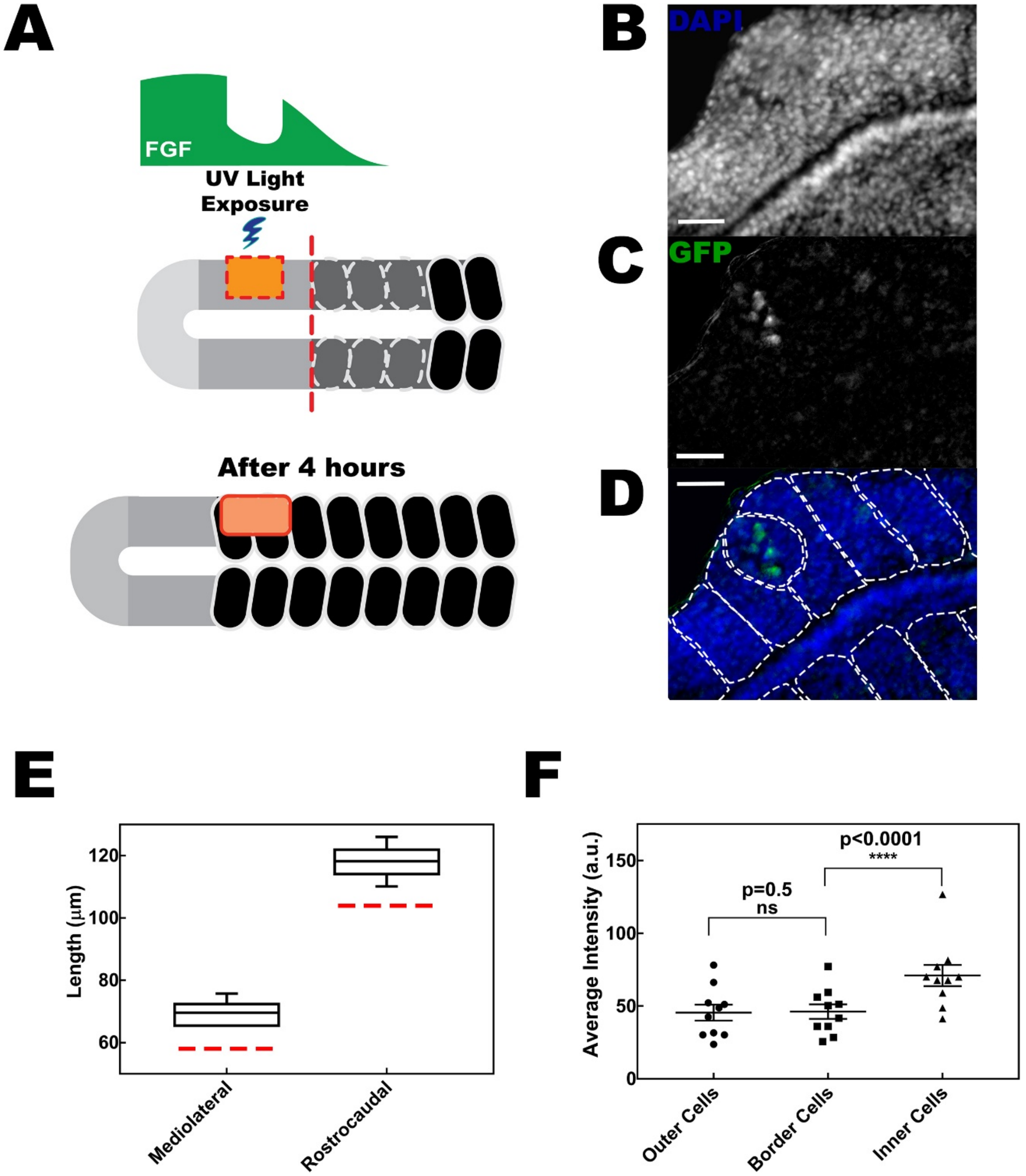

Figure S8. Local Perturbation of FGF Gradient Results Formation of Artificial Somites with Neighboring Cells, Related to Figure 7

(A) Sketch of local heat-shock experiments. UV light exposure of a window of cells (orange rectangle) in the undetermined posterior PSM results a well-like drop of FGF signal gradient (green) due to local heat-shock induced in the *hsp70l:dnfgfr1a-EGFP* transgenic line. Cells effected from the local heat-shock group together and form a precocious somite (bottom). (B-D) Representative nuclear (B) and heat-shock induced GFP (C) and overlaid (D) images of the mesoderm tissue. A circular precocious somite is formed (bottom, overlay) by cells effected from the heat-shock and their peripheral neighbors. (E) Measurement of precocious somite sizes for mediolateral (left, n=7) and rostrocaudal (right, n=14) alignments of rectangular pinhole used in the local heat-shock. Red dashed lines show the sizes of the pinhole measured directly through the microscope. (F) Maximum intensity projection of z-section GFP images (n=10) are quantified in three masks as cells outside the precocious somite (left, outer cells), cells at the internal periphery of the somite (middle, border cells) and cells inside the somite (right, inner cells). Signal from the internal border cells are not significantly different (paired t-test) from the background signal of outer cells. Error bars indicate s.d. Posterior is left.

Table S1

Parameter description	Symbol	Value / Range of values / Calculation
Setup parameters		
Lattice step size		1 cell
Simulation box (1-D)		85 cells
Total simulated time		18 hours (31 clock cycles)
Simulation time unit	stu	30 secs (70 stu=1 clock cycle)
Runge-Kutta time unit		0.3 secs (1 STU=100 iterations)
Size of tail bud		15 cells
Cell diameter		8 μm
Tail bud elongation speed	V_g	1.143 μm/min / variable array / zero for NG
Time delay parameters		
LIG translation time delay	trlatedel	7 mins
SIG activation time delay	actdel	7 mins
INH activation time delay	reprdel	14 mins
Held parameters		
mLIG transcription rate	k_{mLIG}^{syn}	$0.6\ s^{-1}$ (zeroth order)
LIG translation rate	k_{LIG}^{syn}	$0.06\ s^{-1}$ (first order)
SIG translation rate	k_{SIG}^{syn}	$1.2\ s^{-1}$ (zeroth order)
INH translation rate	k_{INH}^{syn}	$0.012\ s^{-1}$ (second order)
mLIG degradation rate	k_{mLIG}^{deg}	$0.0012\ s^{-1}$ (first order)
LIG degradation rate	k_{LIG}^{deg}	$0.012\ s^{-1}$ (first order)
COMP degradation rate	k_{COMP}^{deg}	$0.024\ s^{-1}$ (first order)
SIG degradation rate	k_{SIG}^{deg}	$0.012\ s^{-1}$ (first order)
INH degradation rate	k_{INH}^{deg}	$0.12\ s^{-1}$ (first order)
Variable parameters		
Ligand diffusion speed	D	$3-30$ μm/min (0.2 cell/stu step)
Ligand receptor binding rate	k_{LIG}^{bin}	$0.0012-0.012\ s^{-1}$ (second order)
SIG^- activation rate	k_{SIG}^{act}	$0.0018-0.018\ s^{-1}$ (second order)
SIG^+ deactivation rate	k_{SIG}^{deact}	$0.0018-0.018\ s^{-1}$ (second order)

Table S1. Description of Simulation Parameters, Related to Figure 3
