# Supplementary material for: Spatial Fold Change of FGF Signaling Encodes Positional Information for Segmental Determination in Zebrafish
Source: Cell Rep. Author manuscript; Available in PMC 2018 Jul 27. (PMC6063364; doi:10.1016/j.celrep.2018.06.023)
Supplement: 2 [file NIHMS981694-supplement-2.pdf]

Cell Reports

Article

Spatial Fold Change of FGF Signaling Encodes Positional Information for Segmental Determination in Zebrafish

Graphical Abstract

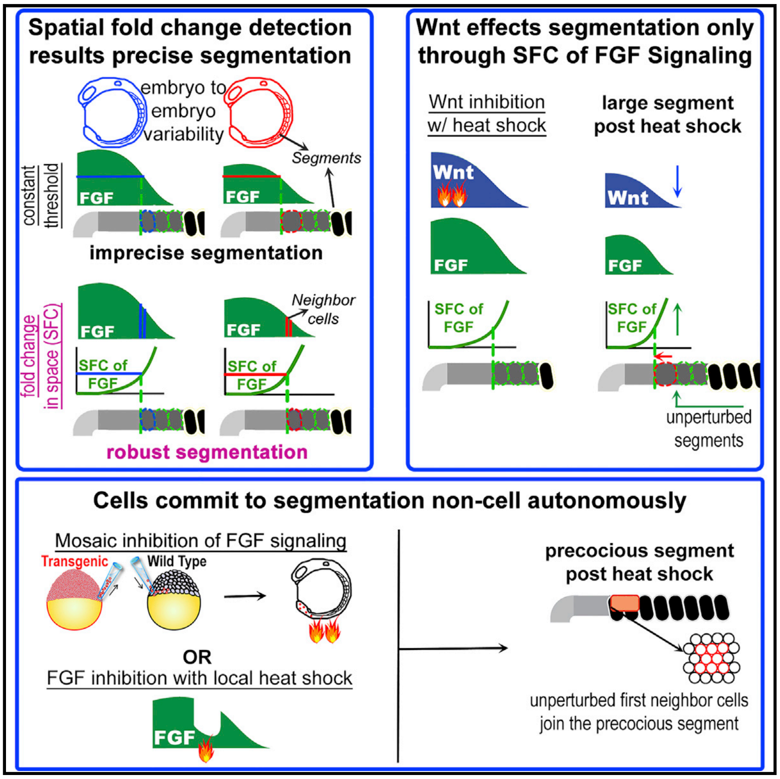

Highlights

- A 3D explant system is used to study mechanisms underlying somite segmentation
- Spatial fold change in FGF signal encodes positional information for pattern formation
- Neighboring cells compare their FGF signal strengths to position segment boundary
- Wnt signaling acts permissively and hierarchically upstream of FGF signaling

Authors

M. Fethullah Simsek, Ertuğrul M. Özbudak

Correspondence

ertugrul.ozbudak@cchmc.org

In Brief

Simsek et al. use an elongation-arrested 3D explant system, integrated with quantitative measurements and computational modeling, to show that positional information for segmentation is encoded solely by spatial fold change (SFC) in FGF signal output. Neighboring cells measure SFC to accurately determine somite segment sizes. Wnt signaling acts permissively upstream of FGF signaling.

Simsek & Özbudak, 2018, Cell Reports *24*, 66–78
July 3, 2018 © 2018 The Author(s).
https://doi.org/10.1016/j.celrep.2018.06.023

CellPress

OPEN ACCESS
CellPress

Cell Reports
Article

Spatial Fold Change of FGF Signaling Encodes Positional Information for Segmental Determination in Zebrafish

M. Fethullah Simsek[1,3] and Ertuğrul M. Özbudak[1,2,3,4,*]
[1]Division of Developmental Biology, Cincinnati Children's Hospital Medical Center, Cincinnati, OH 45229, USA
[2]Department of Pediatrics, University of Cincinnati College of Medicine, Cincinnati, OH 45229, USA
[3]Department of Genetics, Albert Einstein College of Medicine, Bronx, NY 10461, USA
[4]Lead Contact
*Correspondence: ertugrul.ozbudak@cchmc.org
https://doi.org/10.1016/j.celrep.2018.06.023

SUMMARY

Signal gradients encode instructive information for numerous decision-making processes during embryonic development. A striking example of precise, scalable tissue-level patterning is the segmentation of somites—the precursors of the vertebral column—during which the fibroblast growth factor (FGF), Wnt, and retinoic acid (RA) pathways establish spatial gradients. Despite decades of studies proposing roles for all three pathways, the dynamic feature of these gradients that encodes instructive information determining segment sizes remained elusive. We developed a non-elongating tail explant system, integrated quantitative measurements with computational modeling, and tested alternative models to show that positional information is encoded solely by spatial fold change (SFC) in FGF signal output. Neighboring cells measure SFC to accurately position the determination front and thus determine segment size. The SFC model successfully recapitulates results of spatiotemporal perturbation experiments on both explants and intact embryos, and it shows that Wnt signaling acts permissively upstream of FGF signaling and that RA gradient is dispensable.

INTRODUCTION

Cells use many signaling systems to sense their microenvironment and communicate with each other during development, tissue repair, immunity, and normal tissue homeostasis. How instructive information encoded within a signaling (morphogen) gradient is received by cells is a critical question broadly relevant for biology, bioengineering, and medicine. Here, we studied this important problem in the context of somitogenesis, the embryonic patterning of the vertebral column.

Organisms display characteristic patterns, whose sizes scale with variable tissue sizes. Identifying mechanisms governing pattern formation and scaling during development is a long-standing quest. Vertebrate embryos pattern their major body axis as repetitive somites, which segment from the presomitic mesoderm (PSM) progressively as the tail end of the embryo elongates posteriorly. When somite segmentation goes awry, it results in birth defects (Pourquié, 2011). Somitogenesis is both versatile (different species develop characteristic segment numbers and periodicity) and very precise (within a given species, each individual, regardless of final body size, displays the same segment number and size distribution) (Gomez et al., 2008). Several models have been proposed to explain this extraordinary precision and scaling behavior. The clock and wavefront (CW) model explains periodic and precise segmentation of somites by the interaction of a segmentation clock with a posteriorly moving morphogen gradient in the PSM (Cooke, 1975; Cooke and Zeeman, 1976). Pioneering discoveries of molecular oscillators and signaling gradients support this model (Dubrulle et al., 2001; Dubrulle and Pourquié, 2004; Palmeirim et al., 1997; Sawada et al., 2001). While we identified the signaling pathways involved, we still lack a consensus regarding the fundamental mechanisms that orchestrate segment boundary placement and somite scaling. In the classical CW model, a constant threshold of a posteroanterior fibroblast growth factor (FGF) and/or Wnt signaling gradient sets the wavefront position (the determination front) beyond which cells become time-stamped to segment into somites (Pourquié, 2011) (Figure 1A). The opposing gradients model extends the classical CW model and claims that the determination front emerges at the point where opposing retinoic acid (RA) and FGF/Wnt gradients both reach a critical threshold (Diez del Corral et al., 2003) (Figure 1B). In contrast to these long-distance-acting gradient models, a recent study proposed a short-distance, Turing-type model comprising a cell-autonomous activator and a diffusible repressor with a source at the anterior end of the PSM (Cotterell et al., 2015) (Figure 1C).

The existence of three competing mechanisms encoding the determination front is a reflection of real experimental and modeling challenges. First, coupling of axial elongation of the PSM and regression of the signaling gradients instructing the determination front resembles a classical physics problem of determining the position of a moving object in a moving car, accommodating for changes in their speed over time. Such coupling poses a great challenge for the collection of quantitative data needed to unravel the mechanism encoding positional

 © 2018 The Author(s).
This is an open access article under the CC BY license (http://creativecommons.org/licenses/by/4.0/).

OPEN
ACCESS
CellPress

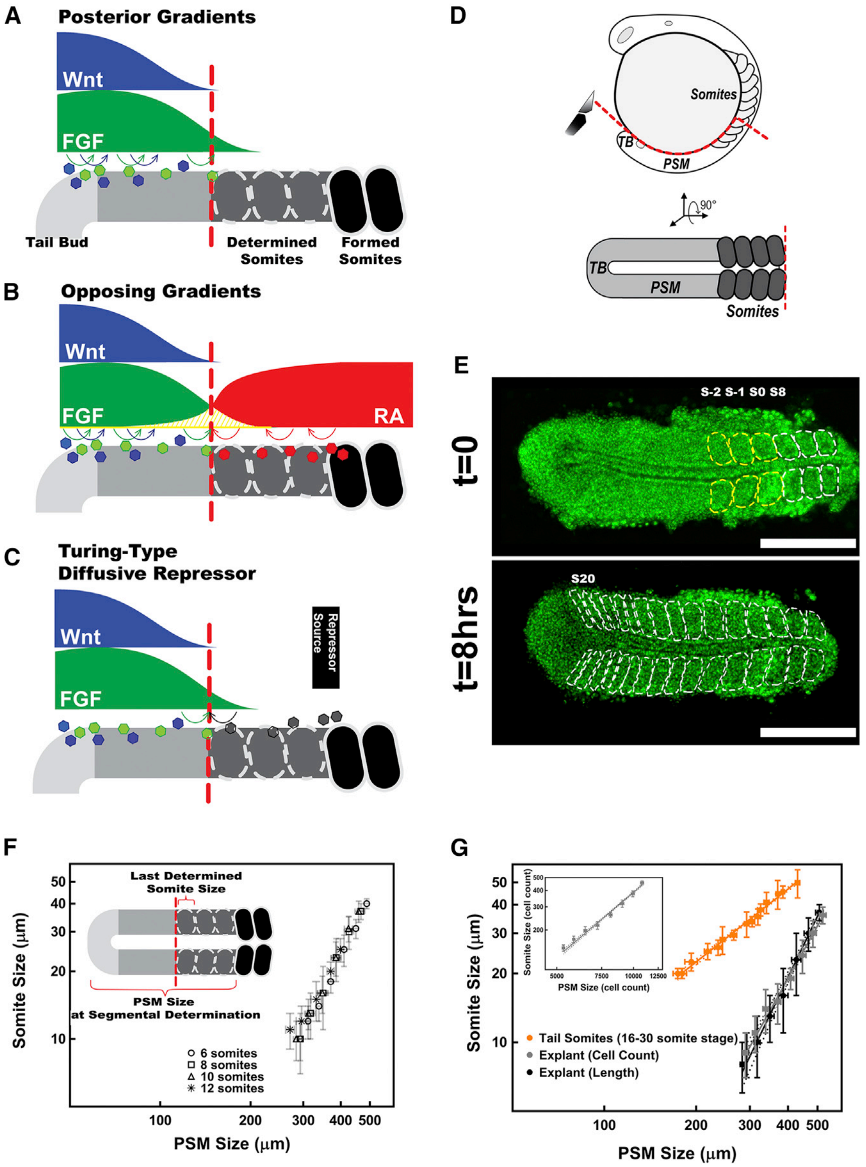

Figure 1. Somitogenesis and Segment Size Scaling

(A–C) Current models explaining how segment sizes are determined: the classical "clock and wavefront" model (A), the "opposing gradients" model (B), and the Turing-type model (C).

(D) Zebrafish embryos (top, lateral view) were cut through red line, and the explants (bottom, dorsal view) were cultured.

(E) A tissue explant (from an 8 somite stage embryo) formed the 3 predetermined somites (yellow dashed lines) followed by 9 progressively smaller somites (white dashed lines) under non-elongation culture over 8 hr. A nuclear-localized GFP marker, in green, was used to count cell numbers. Scale bars, 250 μm.

(F) Somite sizes display the same scaling trend (analysis of covariance [ANCOVA]: $F(4,91) = 0.59$, $p = 0.62$) with the PSM sizes in explants started at different stages ($n = 6, 7, 7$, and 4 for 6, 8, 10, and 12 somite stages, respectively).

(G) The size of tail somites scales with the size of PSM at segmental determination in whole zebrafish embryos (orange, $n = 5$; reanalyzed from Gomez et al., 2008). Length measurement in 1D (black, $n = 9$) and cell counts in 3D (gray, $n = 7$). Cell counts in 3D (insert) are converted to 1D length measurements by geometry of the tissue (STAR Methods).

Data are presented with error bars representing the SDs and linear fit of data in logarithmic scale with 95% confidence bands. Posterior is left.

See also Figures S1 and S2.

information responsible for pattern formation and scaling. Second, it is difficult to disentangle the activities of multiple signaling pathways in the PSM. Third, the integration of quantitative data with predictive molecular-level modeling was lacking in the previous studies. To overcome these challenges and discover the mechanism encoding the determination front, we first developed a 3D zebrafish tail explant model (Figures 1D and 1E) displaying the same scaling of somite sizes observed during formation of embryonic tail somites (Figures 1F and 1G). The explant model effectively decoupled the regression dynamics of signaling gradients from the elongation of PSM. We carried out perturbation experiments using surgical, pharmacological, and local/mosaic genetic activation tools in both explants and whole embryos. These experiments allowed us to disentangle the signaling activities of FGF, Wnt, and RA pathways in the PSM. We then quantified changes in segment size and scaling following each perturbation and computationally modeled the system, generating predictions to be tested experimentally.

The results disfavored variants of the constant-threshold CW models as well as the Turing-type model. Instead, our results showed that cells along the antero-posterior (A/P) axis compare their FGF signaling activity with their neighbors to accurately derive a spatial fold-change (SFC) value and use this information to position the segmental boundary. The SFC of FGF signal encodes positional information; when the SFC exceeds 22%, the neighboring cells commit to place a segment boundary between them. Moreover, we discovered a hierarchical network in which Wnt signaling affected somite pattern formation by acting permissively upstream of FGF signaling.

RESULTS

The 3D Tissue Explant Model Decoupled Regression of the Determination Front from Elongation of the Tail and Unraveled a General Scaling Trend for Segment Sizes

The size of a somite is determined by the distance the determination front regresses posteriorly during each segmentation clock cycle. The regression speed of the determination front

depends both on the speed of axial elongation, which varies throughout somitogenesis (Denans et al., 2015; Gomez et al., 2008), and the regression dynamics of the hitherto-unknown molecular circuit encoding the positional information. To decouple the regression dynamics of the determination front from axial elongation, we developed a 3D PSM tissue explant model (Figures 1D and 1E). Axial elongation is primarily driven by cell ingression into the PSM; we can culture the explants with or without axial elongation leveraging the physical forces applied on the explant adhering to a slide (STAR Methods). This resulted in zero axial elongation (non-elongating explant condition) but did not stall somitogenesis (Figure S1). The segmentation period did not change over the duration of the experiment or in between elongating and non-elongating explants (Figure S1).

Segmental boundary determination occurs in the middle of the PSM. Cells located anterior to the determination front are already predetermined (time-stamped) to segment into somite (Dubrulle et al., 2001; Giudicelli et al., 2007; Sawada et al., 2001). Perturbations of either the segmentation clock (Giudicelli et al., 2007) or the signaling gradients (Dubrulle et al., 2001; Sawada et al., 2001) did not affect the segmentation of these predetermined somites. We first quantified the lengths of the last formed somite and the PSM along the A/P axis, and the number of predetermined somites throughout somitogenesis of whole embryos (STAR Methods; Figures S1). After segmentation of the predetermined somites at the anterior PSM, non-elongating explants formed somites with progressively reduced length (smaller somites, Figure 1E). We observed a scaling trend correlating somite length with PSM length at the time of segmental determination (Figure 1F, insert). Strikingly, this scaling trend was observed in all explants independent of stage or variation in initial PSM length (Figure 1F). This trend accounts for both the change in somite length and the rate of change, described as a slope when somite length is plotted against PSM length (which is reduced by every segmented somite). The decrease in somite size could represent a change in cell number per somite, cell size, or both. We verified that cell size did not change and observed that the scaling was the same whether it was based on explant length measurements or cell number per somite (STAR Methods; Figure 1G, gray). Thus, scaling reflects a progressive change in the position of the determination front.

To test if somite scaling is an explant artifact, we tested the correlation between the previously reported somite and PSM lengths (Gomez et al., 2008) in species of three vertebrate classes (chicken, mouse, and zebrafish). Anterior somites have considerably constant lengths in all three species (Figure S2), whereas posterior somites get progressively shorter; this is because PSM length declines as axis elongation slows down in the posterior (Figures S1E and S1F; Denans et al., 2015; Gomez et al., 2008). We found that the length of a newly formed tail somite decreases in proportion to the length of the PSM at the time of its segmental boundary determination in all three species (STAR Methods; Figure S2). In a living zebrafish embryo, ingressing cells elongate the tail bud, and the scaling slope is shallow (Figure 1G, orange). In non-elongating explants (which do not gain any new cells after entering the culture chamber), the scaling slope is steep (Figure 1G, black and gray). Hence, our explant model uncouples the dynamics of determination front regression from axial elongation and mimics segment size scaling of tail somites in whole vertebrate embryos, and the slope of the scaling trend as well as somite lengths serve as simple readouts that different models for segmental determination should be able to reproduce.

Existing Models Cannot Reproduce the Observed Patterns

Using the scaling trends in the explant model as readout, we first asked if two of the prevailing models of somite segmentation (opposing gradients [Figure 1B] and Turing-type [Figure 1C]) can correctly predict the explant response to perturbations. The sources of the RA gradient in the opposing gradients model and the unknown repressor molecule in the Turing-type model are newly formed somites. Therefore, both these models predict that removal of the newly formed somites and/or the anterior PSM should alter the position of the determination front and hence modify the scaling trends. Whereas control explants contained the whole PSM with 3 to 5 of the most recently segmented somites (Figure 2A, full axis), we imposed two types of anterior dissections as experimental perturbation: PSM tissue without any visible somites but with predetermined somites (Figure 2B, full PSM) and explants with only the posterior PSM without the predetermined somites (Figure 2C, half PSM). Contrary to the models' prediction, we find that all three types of explants exhibited the same ($p = 0.3$) scaling trend (Figures 2D and 2E), arguing against any type of signal provided by tissue anterior to the determination front. Strikingly, the half PSM explants scaled their somite sizes not with their remaining size (half PSM) but with the size that also included the predetermined somites that were dissected out at the beginning of explant culture (Figures 2D and 2E). To rule out residual RA signal, we treated the full axis explants with the RA receptor inhibitor drug BMS493 (50 μM in DMSO) (Figures 2F and 2G). Consistent with a previous study reporting only minor size changes in RA signaling-deficient quail embryos (Diez del Corral et al., 2003), pharmacological inhibition of RA signaling did not change somite sizes in intact zebrafish embryos (Figures S3A and S3B). Of note, RA signaling was perturbed in zebrafish previously, but the impact on somite sizes was not reported (Begemann et al., 2004; Kawakami et al., 2005). The inability of the two models to correctly predict the results of pharmacological and surgical perturbations suggests that a different system determines the position of the determination front (Figures 2A–2G).

We next dissected the PSM tissue into anterior and posterior halves and cultured them in the same well, but without contact with each other (Figures S3C and S3D). Interestingly, we observed that the segmentation of the posterior half of the PSM explants began with a delay of 3 to 4 clock cycles, waiting until after segmentation of its anterior half was completed (Figures S3C and S3D). These results demonstrated that the position of determination front is primarily set by molecules acting posterior to the hypothetical activator-repressor interactions taking place at the anterior end of the PSM proposed by the Turing-type model (Cotterell et al., 2015).

Pharmacological inhibition or ectopic activation of FGF or Wnt signaling pathways altered segment lengths (Aulehla et al., 2003; Dubrulle et al., 2001; Sawada et al., 2001), indicating a shift in the

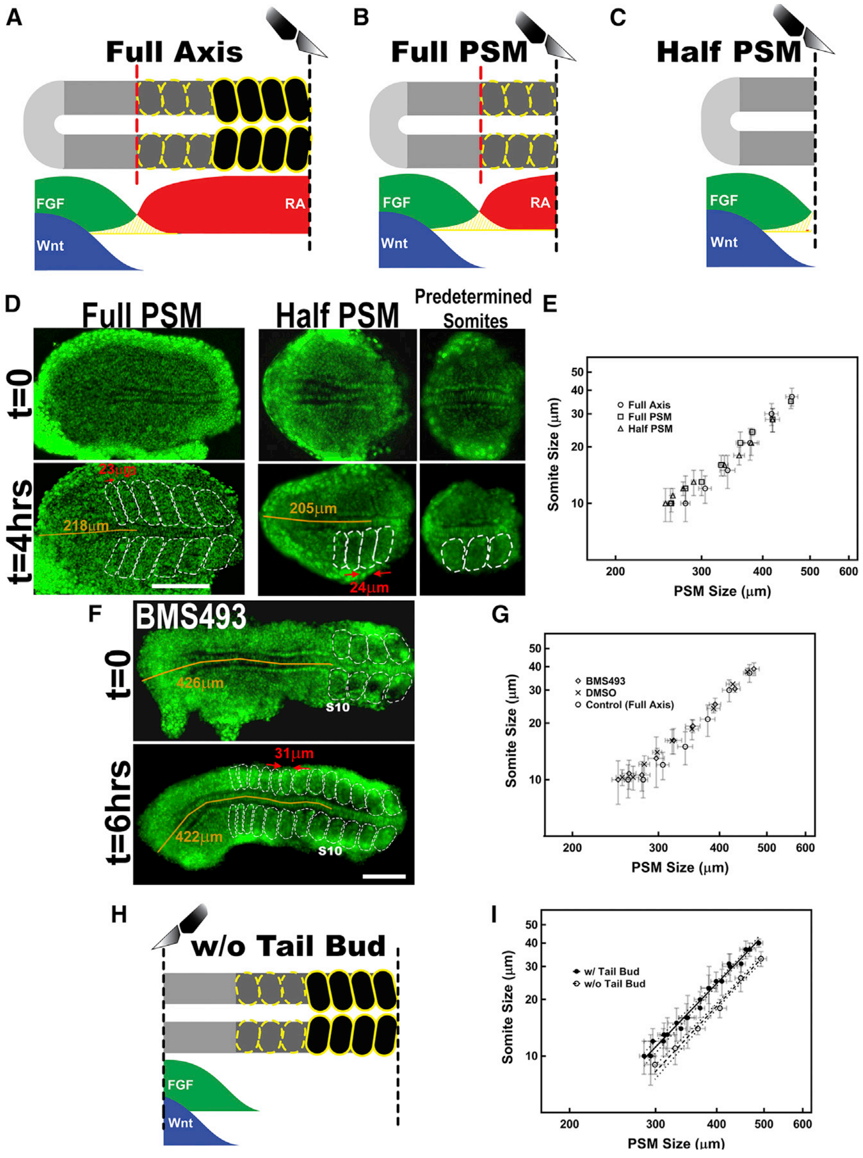

Figure 2. Posterior Morphogen Gradients Provide Positional Information for Segmental Determination

(A–C) Preparing different explants (A, PSM with last formed somites; B, PSM only; and C, posterior half of PSM) alters the effect of anterior RA and/or hypothetical Turing repressor sources.

(D) A full PSM (left) and a half PSM explant (middle) from 10 somite stage embryos formed similar-sized somites at similar PSM coordinates. Images at the beginning of and 4 hr in the explant culture are shown. The predetermined somites, complementary to the half PSM explant, are shown in the right panel. Bright large nuclei flanking lateral to the PSM tissue belong to skin cells.

(E) Full axis (circle), full PSM (square), and half PSM (triangle) explants displayed the same scaling trends (ANCOVA: $F(3,201) = 1.2402$, $p = 0.3$).

(F) A full axis explant from a 10 somite stage embryo was continuously treated with BMS493. Images at the beginning of and 6 hr in the explant culture are shown. The size of the first smaller somite scaling with size of the PSM is marked between red arrows.

(G) Explants treated with BMS493 (diamond, $n = 9$) or DMSO only (cross, $n = 5$) displayed the same scaling trends with untreated full axis explants (circle) (ANCOVA: $F(3,163) = 0.1340$, $p = 0.9$). Error bars indicate SD.

(H) The tail bud is removed to investigate the role of diffusion of posterior gradient ligands in scaling trend.

(I) Scaling trends differed between tailbud-removed (hollow circle, $n = 14$) and control data (full axis, filled circle, $n = 9$).

Posterior is left. See also Figures S1, S3, and S6.

determination front position. The posteroanterior gradients of FGF and Wnt signaling in the PSM (Aulehla et al., 2003; Dubrulle et al., 2001; Sawada et al., 2001) are established in part due to localized transcription of ligands (Dubrulle and Pourquié, 2004), which will automatically be translated into a protein gradient. So far, the role of protein diffusion has not been investigated during somite segmentation. To further assess the roles of the FGF and Wnt diffusion in segmental boundary position, we dissected and removed the tail bud, where FGF/Wnt are transcribed, and tested the impact of removing their transcription zone on the boundary position (Figure 2H). If ligands do not diffuse, then the ligand gradients will not change in the tissue when we remove the transcription zone. Therefore, somite lengths in tail-bud-removed explants will be the same as those in intact explants. Eliminating the FGF/Wnt transcription zone did not change the scaling slope. However, and in contrast to anteriorly dissected explants (Figures 2A–2E), we observed an immediate shift in the position of the determination front as reflected by the formation of shorter somites in posteriorly dissected explants relative to explants containing the tail bud (Figure 2I). After the three predetermined somites segmented, the first somite to be determined following removal of the transcription zone was shorter than equivalent control somites. This result suggests that the signaling morphogens diffuse rapidly and that removal of their transcription zone impacts segment sizes immediately (Figure 2I). Therefore, even though it is possible to establish a posterior gradient merely with the decay of cell-intrinsic RNA levels (Dubrulle and Pourquié, 2004), ligand diffusion is necessary to establish the gradient that provides positional information for segmentation.

To unravel which dynamic feature of the signaling gradients encodes position information, we built a generic mathematical model describing the system. The model incorporates ligand (FGF or Wnt) diffusion, degradation and receptor binding, receptor activation of intracellular signals (double phosphorylation of extracellular receptor kinase ERK [ppERK] or nuclear localization of

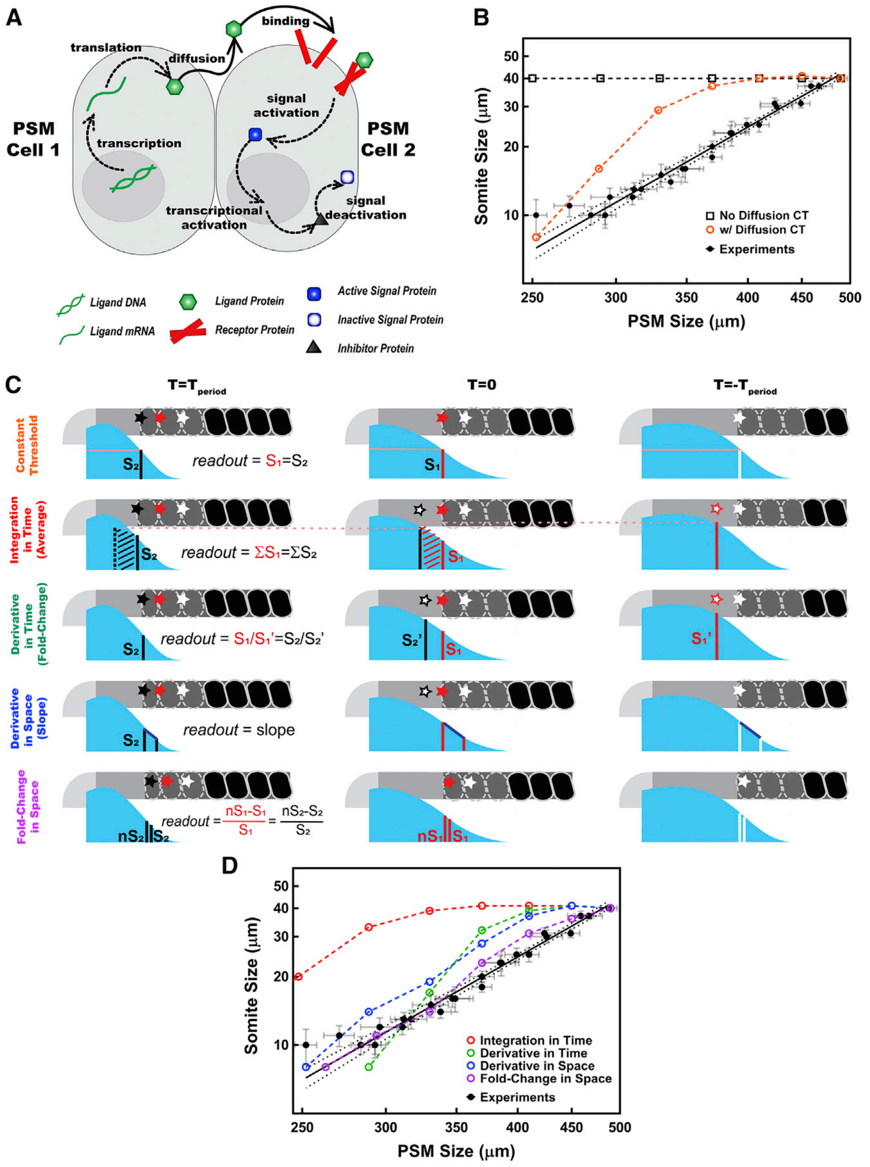

Figure 3. Scaling Phenomena Is Explained *In Silico* by the SFC of Cell Signaling

(A) Core posterior signaling network. Only posterior cells transcribe ligand mRNA. Diffusing ligands (green) bind to receptors (red). Formed complex activates the cascade of signal output protein (blue). Activated signal protein (ppERK or nuclear β-catenin) triggers synthesis of its own inhibitor (black). Inhibitor protein in turn deactivates the signal protein.

(B) Constant-threshold model both with (orange) and without ligand diffusion (black squares) failed to reproduce the scaling trend (black solid line for the logarithmic scale linear fit to pooled data from different stages and dotted line for 95% confidence intervals).

(C) Alternative readout models for encoding positional information. Following the arrest of axial elongation (right), determination front on white cell (star shaped) shifts gradually in two clock cycles (from right to left) and falls on the red and black cells (star shaped) consecutively, while both the PSM and the morphogen gradient (cyan) shrink posteriorly.

(D) Positional information encoding by the SFC (purple), but not the temporal integration (red) nor the derivatives in time (green) and space (blue) of signal recapitulates the scaling trend (black). Posterior is left.

See also Figure S3.

β-catenin), synthesis of inhibitor proteins, and inhibition of signal output (dephosphorylation of ppERK or phosphorylation and nuclear delocalization of β-catenin; Figure 3A; STAR Methods). We restricted transcription of ligand RNA only to cells in the tail bud zone, which reproduced the mRNA gradient observed in the tissue (Dubrulle and Pourquié, 2004). We performed an initial coarse parameter search by simulations of different physiological time delays and reaction rates (STAR Methods; Yu et al., 2009). For each model, we screened 10,000 parameter sets covering a parameter space with 10-fold changes resulting in a wide ranges of signal gradients from extremely steep to extremely shallow with half maximal effective concentration (EC_{50}) positions spanning half the size of PSM. First, we noted that without diffusion in non-elongating explants, each cell would translate the mRNA remaining from when the cell was in the tail bud. In this scenario, the model generated somites of constant length (Figure 3B), which suggested a role for ligand diffusion in line with the conclusion of tail bud dissection experiments (Figure 2I). We further assumed that the morphogen diffuses only anteriorly from the posterior-most cell (a no-flux boundary condition at the posterior), and once the morphogen enters a newly formed somite, it will be completely absorbed by high levels of FGF receptors at the anterior boundary of PSM and endocytosis of receptor/ligand complexes (Scholpp and Brand, 2004). No posterior diffusion will occur (described as perfectly absorbing boundary condition). The simulations revealed that the immediate effect of elongation arrest is to alter the steepness of the gradient rather than changing the signal levels as the determination front approaches the morphogen source. None of the 10,000 permutations positioned the boundary correctly when the model assumes a constant threshold readout; i.e., somite length did not immediately decrease after the arrest of axial elongation (Figure 3B). Hence, each of three prevailing models in the field (Figures 1A–1C) failed to reproduce the observed segment scaling trends (Figures 2 and 3).

A Spatial Fold Change of Signaling between Neighboring Cells Encodes Positional Information and Thereby Determines Segment Sizes

As the constant-threshold CW model prediction (Figure 3B) did not reproduce the observed scaling pattern, we tested four

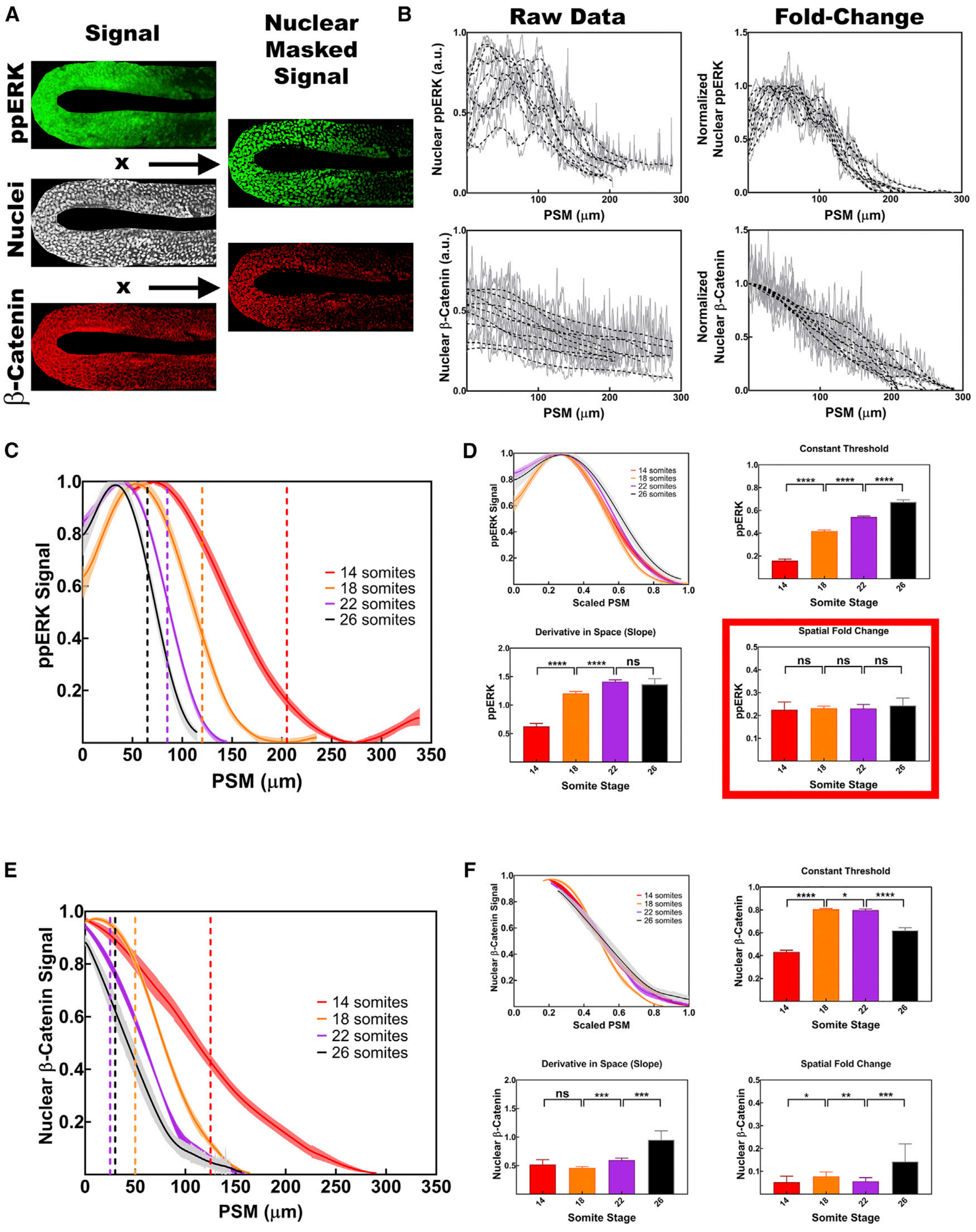

Figure 4. Fold Change of FGF Signaling Is Conserved at the Determination Front

(A) Staining of ppERK (green), β-catenin (red), and Hoechst 33642 (white, used as nuclear mask) at a single z section in the PSM of a 14 somite stage embryo. Nuclear staining of ppERK (green) and β-catenin (red) are shown on the right column.

(B) Embryo-to-embryo variability of signal gradients are significant (left column), whereas normalized gradients (right column) provide a robust signal indicating a fold-change readout mechanism. Raw data (gray lines) are smoothened (black dashed lines).

(C) Normalized levels of ppERK (n = 17, 9, 14, and 13 for 1-4, 18, 22, and 26 somite stages) quantified as FGF signaling output. Posterior end of the PSM are matched for all stages. Determined-undetermined PSM borders are marked by dashed vertical lines with stage matching colors, respectively. Shaded regions represent SEM of data.

(legend continued on next page)

alternative readout models for the positional information encoded by FGF and/or Wnt signaling output: (1) temporal integration of signaling levels (Ben-Zvi et al., 2008; Dessaud et al., 2007; Inomata et al., 2013) of ppERK or β-catenin^{nuclear} (Figure 3C, second row), (2) temporal derivative measuring how fast signaling changes (Figure 3C, third row; Cohen-Saidon et al., 2009; Goentoro and Kirschner, 2009; Wartlick et al., 2011), (3) spatial derivative measuring signal differences between neighboring cells or measuring the slope of gradient (Figure 3C, fourth row; Rogulja and Irvine, 2005), or (4) SFC measuring signaling levels difference between neighboring cells, where each cell computes the percentage difference of signaling between itself and its neighbors (Figure 3C, fifth row). We again screened 10,000 parameter sets for each of the four alternative models. While the SFC model provided multiple parameter sets that recapitulated the observed data, none of the other models could produce any parameter set that fits the observed data (the closest simulations are shown in Figures 3D and S3E).

The SFC model produced two critical predictions. First, fold-change detection should provide robustness against amplitude changes (Goentoro and Kirschner, 2009). To directly test this prediction, we first quantified ppERK and β-catenin^{nuclear} levels by performing immunohistochemistry at 14 somite stage embryos (Figure 4A). Plotting the raw data revealed substantial variability in the ppERK and β-catenin^{nuclear} levels from embryo to embryo (Figure 4B, left column). In the presence of signaling variability, constant threshold readout would not determine segment boundaries accurately in all embryos. On the other hand, normalizing the ppERK and β-catenin^{nuclear} levels to their maximum values in each embryo effectively eliminated the variability in the gradients (Figure 4B, right column). The SFC model automatically achieves such normalization by detecting fold change, not absolute levels. Second, the SFC model predicted that the determination front at all somite stages occurs when the fold differences in signal outputs between neighboring cells reach a fixed value. To test this prediction, we graphed the data for ppERK and β-catenin^{nuclear} levels from 14, 18, 22, and 26 somite stage embryos, aligning all at the posterior end of notochord (Figures 4C and 4E). The PSM border was precisely determined by the position of nuclei, and the position of the determination front was estimated by subtracting the length of the predetermined somites from the length of the PSM (Figure S2) at each stage (Figures 4C and 4E, vertical dashed lines; STAR Methods). The position of the determination front, determined independently of staining, was not aligned with the level of either ppERK or β-catenin^{nuclear} (as would be predicted by the constant threshold model) or by the slope of signal strength (as would be predicted by the spatial derivative model) (Figures 4D and 4F, bar graphs). However, at all stages, the fold change in ppERK signal intensity was fixed at ~22% difference between neighboring cells at determination front positions (Figures 4D and S4A). The same was not true for β-catenin^{nuclear} localization (Figure 4F). This observation validated a critical prediction of the SFC model. The SFC, but not absolute levels of ppERK, was conserved at the determination front even at early stages (3–12 somites) (Figures S4B and S4C), when segment sizes did not scale with PSM size (Figure S2E). Universal conservation of the SFC detection throughout somitogenesis, independent of the scaling phenomenon, suggests that the SFC of FGF signaling encodes positional information, whereas Wnt signaling does not play an instructive role in segmental determination.

Scaling of morphogen gradients with system size has previously been observed in different systems (Ben-Zvi et al., 2008; Inomata et al., 2013). We then replotted the ppERK and β-catenin^{nuclear} levels by normalizing the tissue length at different stages. We observed a scaling of both FGF and Wnt gradients from different stage embryos with the PSM length when we excluded the tail bud from the calculation (Figures 4D and 4F, left top graphs). While neither ppERK nor *fgf8* mRNA gradients scaled with PSM length at anterior somite positions, we have shown that after the 14 somite stage, the scaling of the ppERK gradient mirrored the scaling of the *fgf8* mRNA gradient (Figures S4G and S4H). We posit that since *fgf8* mRNA and ppERK gradients were constant in more anterior somites (Figure S4G), somite length was also constant and did not scale with PSM length at early stages (Figure S2F).

We then asked whether constant threshold readout of scaled gradients could encode positional information. Several observations argued against this: (1) A perfectly scaling gradient encoding positional information predicts that the position of determination front (marking the junction of posterior PSM and pretermined somites) should be kept at a fixed relative position in the PSM. However, the ratio of posterior PSM to full PSM was not kept constant throughout somitogenesis (Figure S1; Gomez et al., 2008). Therefore, this prediction was incorrect. (2) At the determination front position, different gradient readouts are observed at different stages (Figures 4D and 4F, bar graphs; Figure S4B); this variability contradicts a simple constant threshold readout, whereas the fold-change detection mechanism does provide a constant value at every determination front (Figures 4B and S4C). (3) Variability in signaling gradients prevents a simple constant threshold readout and necessitates fold-change detection mechanism (Figure 4B). (4) Even if signal gradient variability was ignored, the constant threshold model cannot explain the scaling trends in explants (Figure 3B). (5) Reducing the PSM by half in explants resulted in somites scaling relative to the initial size of the PSM, not to the size of remaining explant (Figure 2E). A constant threshold readout of

(D) FGF signal gradient for different stages scales with the size of the tissue between posterior tip of notochord and anterior end of the PSM indicating a source and sink mechanism. Readouts of FGF signal outputs according to constant threshold, derivative in space, and SFC between neighboring cells. At the determination front (vertical dashed lines in left panels), the SFC of ppERK reads out precisely the same level (22% ± 2%) at different stage embryos.

(E) Normalized levels of nuclear β-catenin (n = 14, 12, 11, and 11 for 14, 18, 22, and 26 somite stages) quantified as Wnt signaling output. Determined-undetermined PSM borders for Wnt signaling are marked by dashed vertical lines with stage matching colors, respectively.

(F) Similar to ppERK gradient, nuclear β-catenin gradient scales with the tissue size for different stages. Readouts of Wnt signal outputs according to constant threshold, derivative in space, and SFC between neighboring cells.

Error bars represent SEM. Posterior is left. See also Figures S1, S2, and S4.

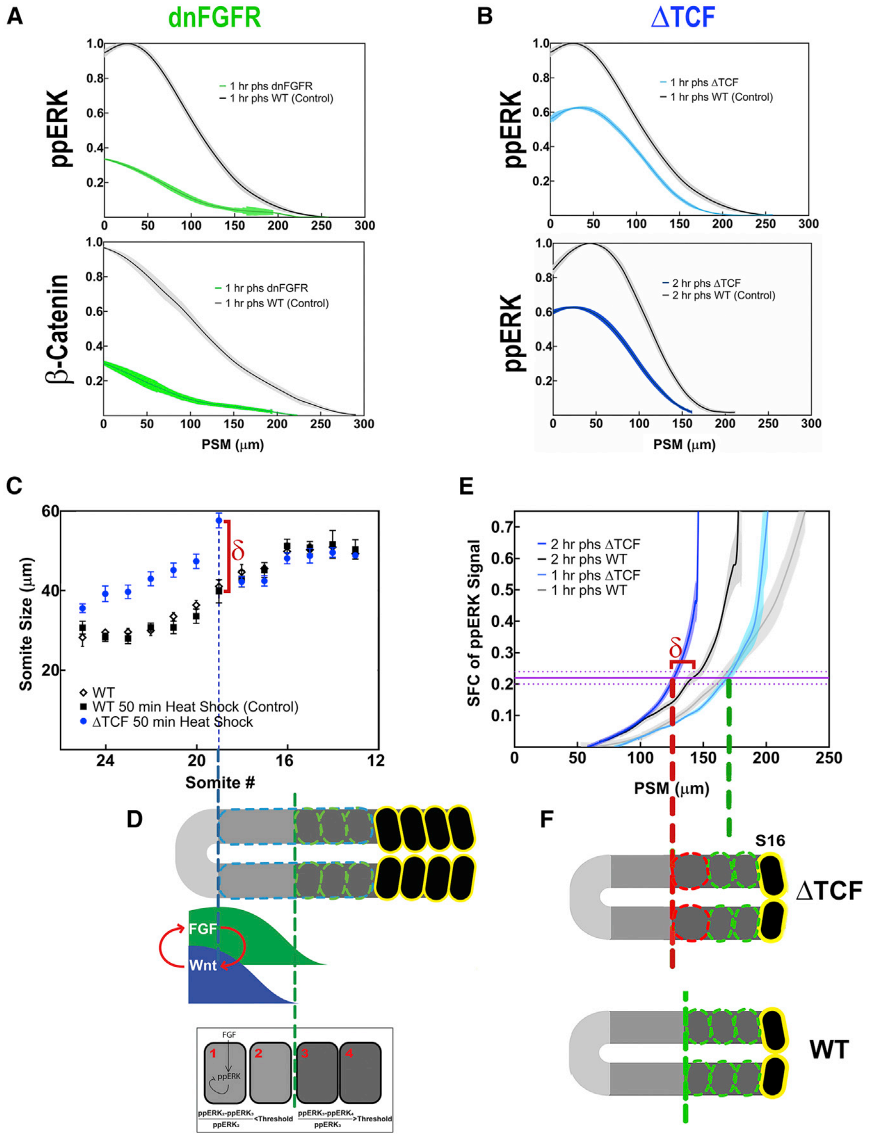

Figure 5. Wnt Signaling Acts Permissively Upstream of FGF Signaling for Segmental Determination

(A) Levels of ppERK (top) and nuclear β-catenin (bottom) following 50 min heat shock perturbation of FGF signaling (green) in comparison with control data for the same stage (black).

(B) Levels of ppERK following 50 min heat shock perturbation of Wnt signaling (blue) in comparison with control data for the same stages (black) over a time course of 2 hr.

(C) Change of somite sizes following the heat shock inhibition of Wnt signaling from 12 to 14 somite stages.

(D) Wnt signaling acts upstream of FGF in segment sizes as a permissive cue in the posterior PSM.

(E) SFC of FGF signaling shifts 2 hr after heat shock posteriorly due to the inhibition of Wnt signaling.

(F) Shift of SFC readout of FGF signaling (δ) matches with the increase of somite size due to Wnt inhibition (C).

Error bars indicate SEM. Posterior is left. See also Figures S1, S4, and S5.

a perfectly scaled gradient requires fine-tuning by adding multiple new assumptions to explain this result. Altogether, our data suggest that segmental determination and segment size scaling are not achieved simply by the scaling of gradients themselves.

Wnt Signaling Acts Permissively Upstream of FGF Signaling

Perturbation of Wnt signaling can change segment sizes (Aulehla et al., 2003; Bajard et al., 2014). We reasoned that if Wnt signaling does not encode the positional information, it might instead act permissively upstream of FGF signaling. We next investigated the hierarchy between the FGF and Wnt signaling pathways in the PSM. We used heat-shock-inducible transgenic lines (*hsp70l:dnfgfr1a-EGFP*, expressing dominant-negative FGF receptor fused with GFP reporter; Lee et al., 2005) and *hsp70l:tcf7l1a-GFP* expressing dominant-negative *tcf7l1a* (TCF) fused with GFP reporter; Lewis et al., 2004) to inhibit the activities of each signaling pathway in a time-controlled manner (STAR Methods). Upon induction, both transgenes (*dnfgfr1a-EGFP* and *tcf7l1a-GFP*) accumulated GFP at the same rate (Figure S5A), each decreasing the outputs of both pathways (Figures 5A and 5B), confirming earlier observations in different species that FGF and Wnt signaling reinforce each other (Aulehla et al., 2008; Wahl et al., 2007). However, we noticed that while perturbation of FGF signaling at the 12 somite stage affected somite sizes beginning at the 16th somite (4 somites delay; Figure S2A), perturbation of Wnt signaling at the same stage affected more posterior somites, starting from the 19th somite (7 somites delay; Figure 5C). Thus, PSM cells in a domain 3 somites long became immune to perturbations of Wnt signaling while remaining sensitive to perturbations in FGF signaling. These results demonstrate that Wnt regulates segmental determination more posteriorly (and therefore at earlier time-points) than FGF signaling does (Figure 5D). Based on these observations, we anticipated that the impact of Wnt perturbations on FGF signal SFC would be delayed more than 1.5 hr (the time it takes to generate 3 somites). We then tested this prediction by inhibiting Wnt signaling at a precise time in transgenic embryos. Although the raw ppERK levels changed within 1 hr after the perturbation (Figure 5B), the SFC of ppERK changed after 2 hr, as predicted (Figure 5E). When the 19th somite was predetermined at the 16 somite stage, the SFC of ppERK was shifted posteriorly by 16 ± 2 μm (relative to control; Figure 5E). A corresponding change in the 19th somite length was recorded (18 ± 3 μm; Figures 5C, 5E, and 5F). The same results were

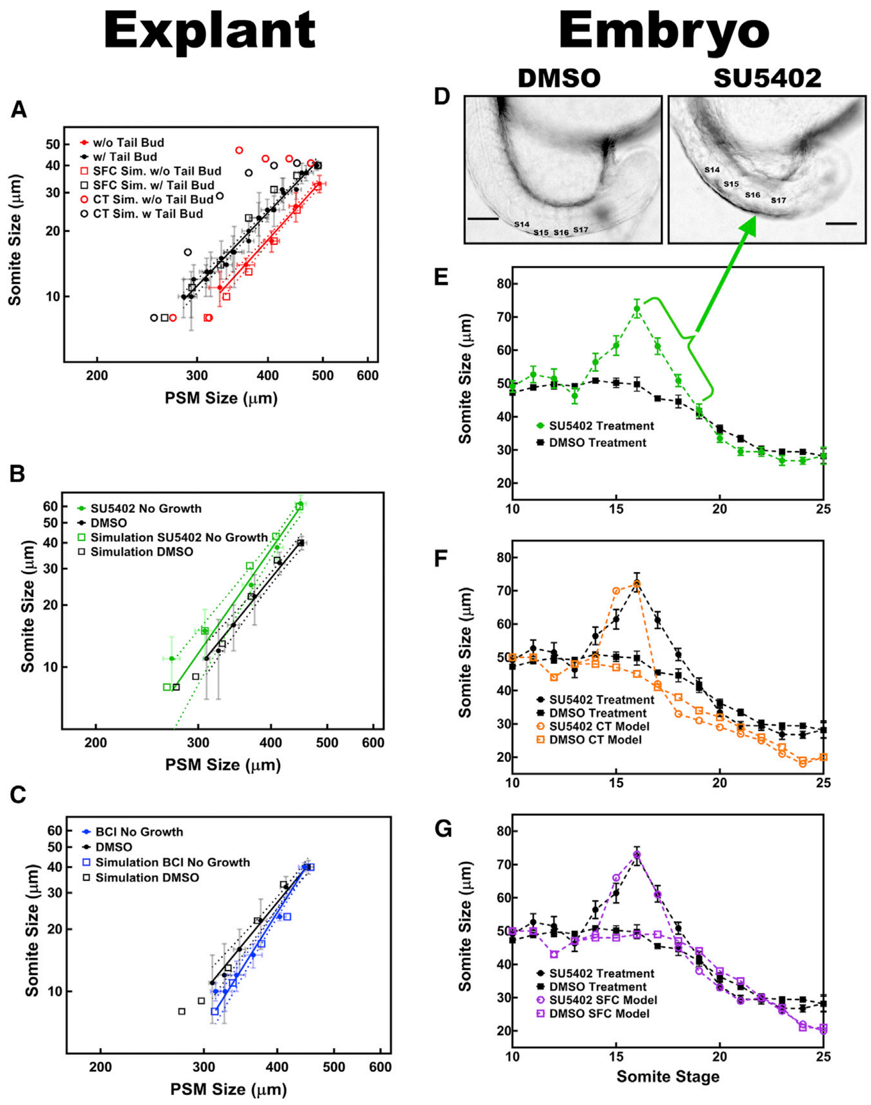

Figure 6. Predictions of the SFC Model Are Validated by Quantitative Experiments in Both Explants and Embryos

(A–C) The SFC, but not the CT, model of FGF signaling successfully predicted changes in scaling trends after tail bud removal (A; n = 14; DMSO only, n = 9), after 10 μM SU5402 treatment (B; n = 6; DMSO only, n = 6), and after 2 μM BCI treatment (C; n = 5; DMSO only, n = 6) in explants.

(D) Transmission light images of 18 somite stage embryos under continuous 2 μM SU5402 drug treatment showing multiple large somites beginning with the 14th somite.

(E) Change of somite sizes through mid-somitogenesis following continuous treatment with SU5402 (green, n = 6; DMSO only, black, n = 5) starting at the 10 somite stage.

(F and G) The SFC model predicts multiple large somites after somite size peaks at 16th somite stage (G). The constant threshold model could not reproduce this result (F).

Experimental scaling data are shown in solid data points. Simulations, shown in hollow markers with matching colors, recapitulated all data using same parameter set. Posterior is left. See also Figures S1, S3, S6, and S7.

obtained using a different Wnt-inhibiting transgenic line (*hsp70l:dkk1b-GFP*; Figures S5B–S5E). Collectively, the data suggest that the FGF signaling SFC is the long-sought information content positioning the determination front, while Wnt signaling acts upstream of FGF signaling in a permissive manner (Figure 5D).

Predictions of the SFC Model Are Successfully Reproduced by Perturbation Experiments in Both Explants and Whole Embryos

When the tail bud is removed, the gradient should become shallower due to a decrease in posteroanterior flux of ligand proteins. According to the constant-threshold CW model, somite should be longer in tailbud-lacking explants with a shallower gradient (Figure 6A). In contrast with these predictions, somite length decreased when the tail bud was removed (Figure 2I). When we quantified the ppERK levels in surgically manipulated half PSM and explants lacking tail buds, we observed that the absolute levels of ppERK were not conserved at the determination front positions. By contrast, FGF signal SFC values were conserved in each determination front all explants (Figure S6), as they were in embryos throughout somitogenesis (Figures 4 and S4). We next tested whether the SFC model simulations could reproduce the changes of segment sizes seen in explants without tail bud (Figure 2I). We found multiple parameter sets that successfully simulated data from both the intact and dissected explants (Figure 6A). The SFC model explains this counterintuitive observation: the ppERK gradient slope (∼ΔS) becomes shallow because the absolute levels of ppERK (S) decreased at the new PSM posterior end after removal of the tail bud (Figures S6H and S6K). The slope decreases more than the absolute level, and thus the fold change of ppERK (ΔS/S) decreases (Figure S6K). This effect results in shorter (not longer) somites in tail-bud-removed explants (Figure S6I).

We used the *in silico* SFC model to make three more predictions. First, the model predicted formation of larger somites with a steeper decline in sizes when FGF signaling is inhibited (Figure 6B). To test this prediction, we treated full axis PSM explants with the FGF receptor (FGFR) inhibitor drug SU5402 (10 μM in DMSO). Indeed, SU5402 treatment resulted in larger somites followed by a steeper scaling trend (Figure 6B). Second, the simulations predicted formation of even smaller somites with a steeper scaling trend if the negative feedback loop in the signaling network was weakened (Figure 6C). To test this prediction, we targeted DUSP1/6, which is downstream of FGF

signaling and inhibits the activity of FGF signaling by dephosphorylating ppERK. As predicted, treating explants with the DUSP1/6 inhibitor drug BCI (2 μM in DMSO) resulted in steeper decline in scaling trend as compared to DMSO-only treatment data (Figure 6C).

We next tested the third SFC model prediction in whole embryos. We treated zebrafish embryos continuously with a low dose of SU5402 (2 μM) that did not change the tail elongation speed (Figure S7A), starting at the 10 somite stage. When embryos were treated with low doses of SU5402, it took a while for ppERK to attain a new, lower level that was kept constant afterward and regresses with the speed of tail elongation (which was not affected with low SU5402 treatment). Following normal segmentation of the 3 predetermined somites, we observed formation of 4 or 5 large somites before the sizes of the following somites were reduced back to control sizes (Figures 6D and 6E). The longest somite was formed after two segmentation cycles (Figure 6E) due to the slow rate of change in ppERK levels (Figures S7B and S7C). Both SFC and the constant-threshold CW model were able to model the increase in somite length until it reached its maxima by simulating the gradual effect of drug over two segmentation cycles. Once at steady state, the ppERK profile did not change further and the constant-threshold CW model (which only looks at the level [S]; Figure S6K) predicted incorrectly an immediate return to a somite length set by the tail elongation speed (which is not affected by low SU5402 treatment). By contrast, the SFC model computed $\Delta S/S$ and correctly predicted several large somites forming before somite sizes decrease back to normal (Figure 6G), consistent with the experimental observations. The accuracy of the SFC model was confirmed by the observation that a fixed SFC value (~22%) of FGF signaling output was measured at all determination fronts throughout somitogenesis in intact embryos and explants (Figures 4D, 6, S4, and S6) and following Wnt (Figures 5 and S5) and FGF inhibition (Figures 6 and S7).

Mosaic Experiments Confirm the SFC Model's Prediction of Non-cell-autonomous Segmental Determination

A unique feature of the SFC model is that it relies on a non-cell-autonomous signal-encoding mechanism for segmental determination (the SFC depends on levels of ppERK in both of the neighboring cells). To test this aspect of the model directly, we designed an experiment to form precocious segments within the posterior PSM of whole embryos and tested if wild-type cells can be recruited by their mutant neighbors to participate in the somite. We transplanted 40–50 cells from embryos of the *hsp70l:dnfgfr1a-EGFP* transgenic line to the same latitudes of wild-type embryos at the blastula stages (Figure 7A). We then heat shocked 8–12 somite stage host embryos and performed immunostaining to detect ppERK and GFP in the posterior PSM (Figures 7B–7D). Our results showed that the levels of ppERK dropped cell autonomously only in transgenic GFP-positive cells, but not in their immediate neighbors (Figure 7E). Simulations of all other readout models predict that only dominant-negative FGFR (dnFGFR)-expressing cells in which FGF signaling output is low would precociously commit to segmentation (Figure 7F, top). Conversely, the SFC model predicts that the neighbors of dnFGFR-expressing cells will be recruited to join the precocious segments (Figure 7F, bottom). We then performed immunostaining on host embryos, which were fixed 4 hr after heat shock, and observed formation of irregularly shaped and enlarged somites (Figures 7G–7I). GFP staining detected GFP-positive central cells surrounded by a ring of GFP-negative cells within ectopic somites (Figures 7J–7N), validating the prediction of the SFC model.

We next tested this prediction in 3D explants. We applied local heat shock using pinhole-restricted light exposure to the tissue explants (Figure S8A) from the *hsp70l:dnfgfr1a-EGFP* transgenic line to cell autonomously block FGFR activity in specific groups of cells. We performed cell nuclei staining (Figure S8B) and immunohistochemistry against GFP (Figure S8C) to precisely identify cells forming the precocious somites and cells affected by the local heat shock, respectively (Figure S8D). We observed formation of precocious somite in a domain larger than that expressing dnFGFR-GFP (Figure S8E). We further quantified the average dnFGFR-GFP intensity from cells outside of the precocious segments (background level), cells at the border of segments, or cells in the interior of the segment. Our analysis showed interior cells expressed GFP, but border cells did not (Figure S8F), indicating that cells that did not express dnFGFR-GFP were recruited to the somite, as was uniquely predicted by the SFC model (Figure S8F). These results in both whole embryos and explants demonstrate that cells commit to segmentation by comparing their FGF signaling output to that of their neighbors.

DISCUSSION

The exact mechanism positioning the determination front was a contested subject prior to this study. Variants of the long-standing CW model proposed that positional information was encoded by a constant threshold of the morphogen gradient (Cooke and Zeeman, 1976; Diez del Corral et al., 2003; Pourquié, 2011). A more recent Turing-type model challenged that view and attributed segmental determination to a yet-to-be-discovered diffusible molecule (Cotterell et al., 2015). To resolve this conflict and uncover which mechanism positioned the somite boundary, we developed a novel 3D PSM explant model that decoupled axis elongation from the patterning mechanism, performed spatiotemporal perturbation experiments (both in explants and in whole embryos), and carried out quantitative measurements and computational modeling based on well-defined molecules involved in the signaling cascade known to affect the somite. This parsimonious modeling approach was used to test the fit of current models to the results of multiple perturbation experiments. Surprisingly, none of the three prevailing models (constant morphogen thresholds, opposing gradients, and Turing instabilities) produce the observed response to perturbations. This necessitated a search for an alternative model, which resulted in the discovery of a paradigm-shifting information-encoding mechanism measuring the SFC in FGF signal output. The SFC model showed that when neighboring cells measure a difference of 22% between them in the output of FGF signals, they position a boundary between them under all the conditions we tested. Moreover, experimental

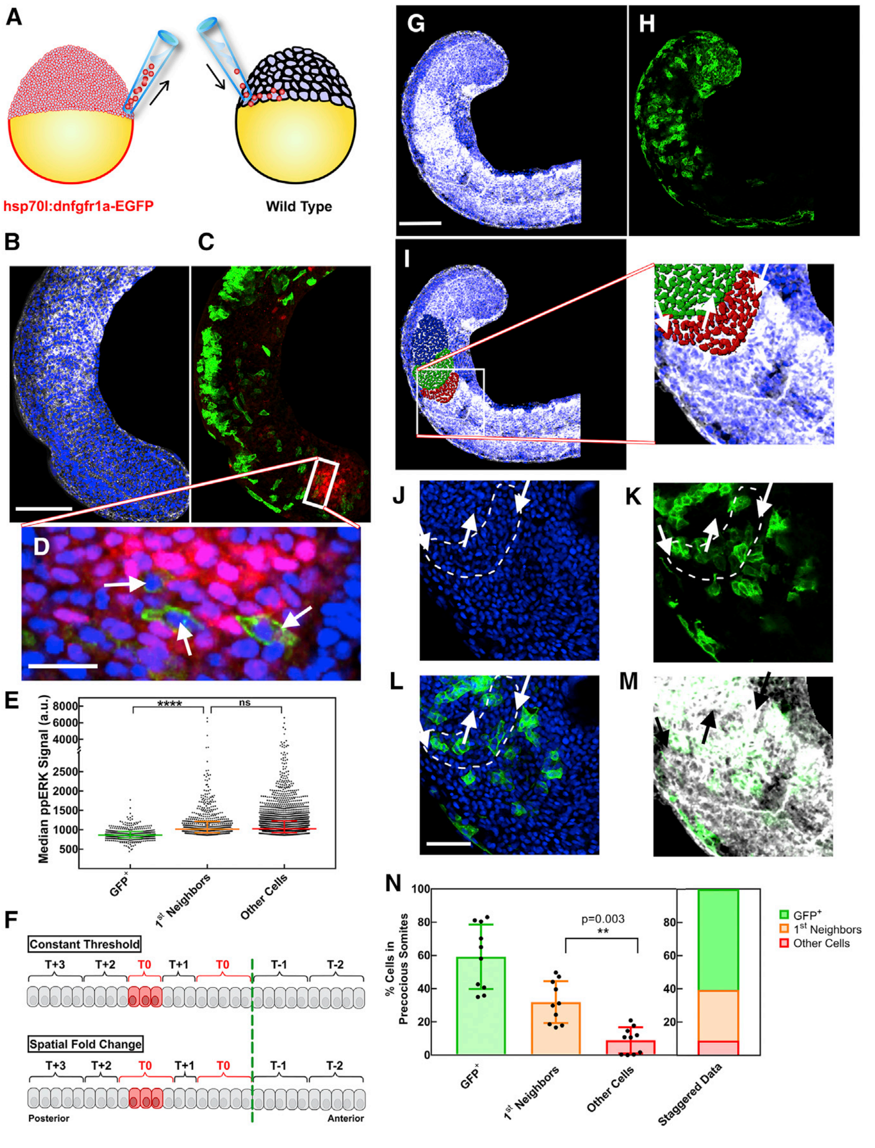

Figure 7. Mosaic Inhibition of FGF Signaling in the PSM of Whole Embryos Results in Formation of Precocious Somites Involving Wild-Type Cells

(A) Sketch of transplantation experiment. Cells responsive to heat shock (red) are transplanted from donor embryo to wild-type host embryo (black) in mid-blastula stage, targeting a specific region.

(B) Nuclear (blue) and fibronectin (white) staining of host embryo tail fixed 4 hr after heat shock. Scale bar, 100 μm.

(C) Immunostaining shows GFP^+ transgenic cells affected from heat shock (green) and ppERK expression as FGF signaling output in the posterior PSM (red).

(D) Zoomed confocal z section image from the tail bud (posterior end of ppERK expression domain). FGF signaling dropped in GFP^+ cells (arrows), whereas their wild-type neighbors were not affected. Scale bar, 20 μm.

(E) Quantification of median ppERK intensity of GFP^+ cells (n = 378, green) located in the posterior ppERK domain of embryos (n = 32) in comparison with their first-neighbor wild-type (orange) and the rest of the cells (red).

(F) In simulations, cells are bracketed in groups as they are determined to form a somite at the end of each segmentation cycle. Heat shock is applied within the 0th cycle to only three PSM cells (20–22, red). All readout models predict determination of a precocious somite cell autonomously at the end of 0th cycle (red). Diverging from others, the SFC model predicts neighboring cells to join the precocious somite due to neighbor comparison (bottom).

(G) Fibronectin (white) and nuclear (blue) staining showing formation of large and irregular somites following heat shock. Scale bar, 100 μm.

(H) GFP^+ cells located posterior to the determination front during heat shock trigger formation of these somites.

(I) Cells within these somites were identified and color-coded with Imaris software by tracing fibronectin deposition at the boundaries.

(J) Zoomed image from (G)–(I) shows spatial organization of cell nuclei at the boundaries of anterior-most precocious somite.

(K) GFP^+ cells are surrounded by a layer of wild-type cells in the precocious somite (arrows).

(L) Overlay of (J) and (K). Scale bar, 40 μm.

(M) Overlay of (K) with fibronectin staining (white).

(N) Cells in precocious somites from different embryos (n = 10) were classified as GFP^+ cells (green), their wild-type first neighbors (orange), and wild-type cells that are not neighboring transgenic cells. A significant wild-type neighbor contribution was observed in precocious somites (>30%). More than 90% of cells forming precocious somites were either transgenic or their first neighbors.

See also Figure S8.

perturbations designed to specifically test SFC-model-predicted phenomena in intact and perturbed tissues validated the model.

Previous work attributed an instructive role for Wnt signaling (Bajard et al., 2014) but did not quantify how the SFC of ppERK changes upon inhibition of Wnt signaling, which prevented them from monitoring the information-encoding aspect of FGF signals. By observing the time delay of perturbation in each pathway, we were able to disentangle the contributions of FGF and Wnt signaling gradients and show that Wnt signaling acts permissively upstream of FGF signaling, which encodes the information used to influence segment length.

ppERK accumulation is more dynamic in mice (Niwa et al., 2011) than in zebrafish (Akiyama et al., 2014; Bajard et al., 2014; Sawada et al., 2001). Despite this species-specific difference, the segmentation clock is necessary for the conversion of spatial signaling readouts into discrete commitment of cells to segmentation at the determination front in all species (Akiyama et al., 2014; Niwa et al., 2011). The conservation of the SFC levels of ppERK at each determination front indicates that the segmentation clock must act in conjunction with the cellular SFC "decoder" (Figures 4D and S4C) and is not monitoring a threshold of ppERK as claimed previously (Akiyama

et al., 2014). Potential decoding mechanisms of a SFC in ppERK could include the planar cell polarity pathway, the Hippo-YAP pathway, Eph-ephrin signaling, and integrin-fibronectin or cadherin-cadherin interactions (Lander, 2011; Rogulja et al., 2008). The molecular mechanism enabling cells to decode the SFC of FGF, and how it integrates with the segmentation clock components, remains to be determined.

Segment size scaling is a phenomenon observed in zebrafish (this report) and in mouse monolayer cell culture in which the scaling is correlated with, and attributed to, the slowing down of segmentation clock oscillations along the A/P axis (phase-difference model; Lauschke et al., 2013). A later study in zebrafish showed that the spatial phase difference of the clock oscillations is not kept constant *in vivo* and argued against the phenomenological phase-difference model (Soroldoni et al., 2014). However, a causal relationship between oscillation phases and segment-length scaling has not been demonstrated, nor has this relationship been tested *in vivo*, until this report. Since FGF signaling has previously been shown to control the spatial phase difference of the clock oscillations (Dubrulle et al., 2001; Sawada et al., 2001), it seemed likely that the FGF signaling gradient controls both segmental boundary determination (somite length scaling pattern) as well as spatial phase difference of the clock oscillations. Alternatively, scaling patterns and spatial phase difference in clock oscillations might simply be correlated with each other.

Various mechanisms were proposed to control pattern scaling in different tissues (Bier and De Robertis, 2015). Our proposed fold-change detection mechanism is analogous to Weber's law in the sensory systems (Goentoro and Kirschner, 2009), except the signal differentiation happens in space rather than in time and is decoded by pairs of cells instead of a single sensory cell. A fold-change detection mechanism provides robustness in signal decoding (output), allowing for variability (noise) in the encoded signal to be filtered out (Goentoro and Kirschner, 2009). FGF and Wnt signaling gradients overlap in many tissues, such as lung epithelium, limb bud, prechordal plate, hindgut, and lateral line primordium (Gibbs et al., 2017; Gros et al., 2010; Dalle Nogare and Chitnis, 2017; ten Berge et al., 2008; Volckaert and De Langhe, 2015). We anticipate that our explant system will be amenable to investigation into the signal decoding mechanisms and our quantitative approach applicable to other tissue patterning systems.

STAR★METHODS

Detailed methods are provided in the online version of this paper and include the following:

- KEY RESOURCES TABLE
- CONTACT FOR REAGENT AND RESOURCE SHARING
- EXPERIMENTAL MODEL AND SUBJECT DETAILS
  - Fish stocks
- METHOD DETAILS
  - PSM Tissue Explants
  - Imaging and Microscopy
  - Pharmacological Treatments
  - Heat shock Experiments
  - Local Heat shock Experiments
  - Cell Transplantation
  - *In Situ* Hybridization
  - Immunohistochemistry
  - Counting Predetermined Somites
- QUANTIFICATION AND STATISTICAL ANALYSIS
  - Signaling Network Model
  - Simulations
  - Determination Front Readouts from Simulations
  - Calculation of PSM Size at Segmental Determination
  - Image Analysis
  - Statistical Analysis
- DATA AND SOFTWARE AVAILABILITY

SUPPLEMENTAL INFORMATION

Supplemental Information includes eight figures and one table and can be found with this article online at https://doi.org/10.1016/j.celrep.2018.06.023.

ACKNOWLEDGMENTS

This work was funded by an NIH grant (GM111987) to E.M.Ö. We thank Florence Marlow and Joshua Waxman for fish strains; Chunyue Yin for transplantation training; Bilge Ugursu, Wesam Azaizeh, and Greg Hamilton for technical help; and Stuart Newman, Ahmet Yildiz, Arthur Lander, Aaron Zorn, and Rafi Kopan for critically reading and assistance in editing (Rafi Kopan) the manuscript.

AUTHOR CONTRIBUTIONS

E.M.Ö. designed and supervised the project. M.F.S. performed the experiments, modeling, and analysis of data. E.M.Ö. and M.F.S. wrote the manuscript.

DECLARATION OF INTERESTS

The authors declare no competing interests.

Received: December 18, 2017
Revised: May 8, 2018
Accepted: June 5, 2018
Published: July 3, 2018

REFERENCES

Akiyama, R., Masuda, M., Tsuge, S., Bessho, Y., and Matsui, T. (2014). An anterior limit of FGF/Erk signal activity marks the earliest future somite boundary in zebrafish. Development *141*, 1104–1109.

Aulehla, A., Wehrle, C., Brand-Saberi, B., Kemler, R., Gossler, A., Kanzler, B., and Herrmann, B.G. (2003). Wnt3a plays a major role in the segmentation clock controlling somitogenesis. Dev. Cell *4*, 395–406.

Aulehla, A., Wiegraebe, W., Baubet, V., Wahl, M.B., Deng, C., Taketo, M., Lewandoski, M., and Pourquié, O. (2008). A beta-catenin gradient links the clock and wavefront systems in mouse embryo segmentation. Nat. Cell Biol. *10*, 186–193.

Bajard, L., Morelli, L.G., Ares, S., Pécréaux, J., Jülicher, F., and Oates, A.C. (2014). Wnt-regulated dynamics of positional information in zebrafish somitogenesis. Development *141*, 1381–1391.

Begemann, G., Marx, M., Mebus, K., Meyer, A., and Bastmeyer, M. (2004). Beyond the neckless phenotype: influence of reduced retinoic acid signaling on motor neuron development in the zebrafish hindbrain. Dev. Biol. *271*, 119–129.

Ben-Zvi, D., Shilo, B.Z., Fainsod, A., and Barkai, N. (2008). Scaling of the BMP activation gradient in Xenopus embryos. Nature *453*, 1205–1211.

Bier, E., and De Robertis, E.M. (2015). Embryo development. BMP gradients: a paradigm for morphogen-mediated developmental patterning. Science *348*, aaa5838.

Campbell, P.D., Chao, J.A., Singer, R.H., and Marlow, F.L. (2015). Dynamic visualization of transcription and RNA subcellular localization in zebrafish. Development *142*, 1368–1374.

Cohen-Saidon, C., Cohen, A.A., Sigal, A., Liron, Y., and Alon, U. (2009). Dynamics and variability of ERK2 response to EGF in individual living cells. Mol. Cell *36*, 885–893.

Cooke, J. (1975). Control of somite number during morphogenesis of a vertebrate, Xenopus laevis. Nature *254*, 196–199.

Cooke, J., and Zeeman, E.C. (1976). A clock and wavefront model for control of the number of repeated structures during animal morphogenesis. J. Theor. Biol. *58*, 455–476.

Cotterell, J., Robert-Moreno, A., and Sharpe, J. (2015). A local, self-organizing reaction-diffusion model can explain somite patterning in embryos. Cell Syst. *1*, 257–269.

Dalle Nogare, D., and Chitnis, A.B. (2017). A framework for understanding morphogenesis and migration of the zebrafish posterior Lateral Line primordium. Mech. Dev. *148*, 69–78.

Denans, N., Iimura, T., and Pourquié, O. (2015). Hox genes control vertebrate body elongation by collinear Wnt repression. eLife *4*, e04379.

Dessaud, E., Yang, L.L., Hill, K., Cox, B., Ulloa, F., Ribeiro, A., Mynett, A., Novitch, B.G., and Briscoe, J. (2007). Interpretation of the sonic hedgehog morphogen gradient by a temporal adaptation mechanism. Nature *450*, 717–720.

Diez del Corral, R., Olivera-Martinez, I., Goriely, A., Gale, E., Maden, M., and Storey, K. (2003). Opposing FGF and retinoid pathways control ventral neural pattern, neuronal differentiation, and segmentation during body axis extension. Neuron *40*, 65–79.

Dubrulle, J., and Pourquié, O. (2004). fgf8 mRNA decay establishes a gradient that couples axial elongation to patterning in the vertebrate embryo. Nature *427*, 419–422.

Dubrulle, J., McGrew, M.J., and Pourquié, O. (2001). FGF signaling controls somite boundary position and regulates segmentation clock control of spatiotemporal Hox gene activation. Cell *106*, 219–232.

Gibbs, H.C., Chang-Gonzalez, A., Hwang, W., Yeh, A.T., and Lekven, A.C. (2017). Midbrain-hindbrain boundary morphogenesis: at the intersection of Wnt and Fgf signaling. Front. Neuroanat. *11*, 64.

Giudicelli, F., Ozbudak, E.M., Wright, G.J., and Lewis, J. (2007). Setting the tempo in development: an investigation of the zebrafish somite clock mechanism. PLoS Biol. *5*, e150.

Goentoro, L., and Kirschner, M.W. (2009). Evidence that fold-change, and not absolute level, of beta-catenin dictates Wnt signaling. Mol. Cell *36*, 872–884.

Gomez, C., Ozbudak, E.M., Wunderlich, J., Baumann, D., Lewis, J., and Pourquié, O. (2008). Control of segment number in vertebrate embryos. Nature *454*, 335–339.

Gros, J., Hu, J.K.H., Vinegoni, C., Feruglio, P.F., Weissleder, R., and Tabin, C.J. (2010). WNT5A/JNK and FGF/MAPK pathways regulate the cellular events shaping the vertebrate limb bud. Curr. Biol. *20*, 1993–2002.

Inomata, H., Shibata, T., Haraguchi, T., and Sasai, Y. (2013). Scaling of dorsal-ventral patterning by embryo size-dependent degradation of Spemann's organizer signals. Cell *153*, 1296–1311.

Kawakami, Y., Raya, A., Raya, R.M., Rodríguez-Esteban, C., and Izpisúa Belmonte, J.C. (2005). Retinoic acid signalling links left-right asymmetric patterning and bilaterally symmetric somitogenesis in the zebrafish embryo. Nature *435*, 165–171.

Lander, A.D. (2011). Pattern, growth, and control. Cell *144*, 955–969.

Lauschke, V.M., Tsiairis, C.D., François, P., and Aulehla, A. (2013). Scaling of embryonic patterning based on phase-gradient encoding. Nature *493*, 101–105.

Lee, Y., Grill, S., Sanchez, A., Murphy-Ryan, M., and Poss, K.D. (2005). Fgf signaling instructs position-dependent growth rate during zebrafish fin regeneration. Development *132*, 5173–5183.

Lewis, J.L., Bonner, J., Modrell, M., Ragland, J.W., Moon, R.T., Dorsky, R.I., and Raible, D.W. (2004). Reiterated Wnt signaling during zebrafish neural crest development. Development *131*, 1299–1308.

Molina, G., Vogt, A., Bakan, A., Dai, W., Queiroz de Oliveira, P., Znosko, W., Smithgall, T.E., Bahar, I., Lazo, J.S., Day, B.W., and Tsang, M. (2009). Zebrafish chemical screening reveals an inhibitor of Dusp6 that expands cardiac cell lineages. Nat. Chem. Biol. *5*, 680–687.

Niwa, Y., Shimojo, H., Isomura, A., González, A., Miyachi, H., and Kageyama, R. (2011). Different types of oscillations in Notch and Fgf signaling regulate the spatiotemporal periodicity of somitogenesis. Genes Dev. *25*, 1115–1120.

Palmeirim, I., Henrique, D., Ish-Horowicz, D., and Pourquié, O. (1997). Avian hairy gene expression identifies a molecular clock linked to vertebrate segmentation and somitogenesis. Cell *91*, 639–648.

Pourquié, O. (2011). Vertebrate segmentation: from cyclic gene networks to scoliosis. Cell *145*, 650–663.

Rogulja, D., and Irvine, K.D. (2005). Regulation of cell proliferation by a morphogen gradient. Cell *123*, 449–461.

Rogulja, D., Rauskolb, C., and Irvine, K.D. (2008). Morphogen control of wing growth through the Fat signaling pathway. Dev. Cell *15*, 309–321.

Sawada, A., Shinya, M., Jiang, Y.J., Kawakami, A., Kuroiwa, A., and Takeda, H. (2001). Fgf/MAPK signalling is a crucial positional cue in somite boundary formation. Development *128*, 4873–4880.

Schindelin, J., Arganda-Carreras, I., Frise, E., Kaynig, V., Longair, M., Pietzsch, T., Preibisch, S., Rueden, C., Saalfeld, S., Schmid, B., et al. (2012). Fiji: an open-source platform for biological-image analysis. Nat Methods *9*, 676–682.

Scholpp, S., and Brand, M. (2004). Endocytosis controls spreading and effective signaling range of Fgf8 protein. Curr. Biol. *14*, 1834–1841.

Soroldoni, D., Jörg, D.J., Morelli, L.G., Richmond, D.L., Schindelin, J., Jülicher, F., and Oates, A.C. (2014). Genetic oscillations. A Doppler effect in embryonic pattern formation. Science *345*, 222–225.

Stoick-Cooper, C.L., Weidinger, G., Riehle, K.J., Hubbert, C., Major, M.B., Fausto, N., and Moon, R.T. (2007). Distinct Wnt signaling pathways have opposing roles in appendage regeneration. Development *134*, 479–489.

ten Berge, D., Brugmann, S.A., Helms, J.A., and Nusse, R. (2008). Wnt and FGF signals interact to coordinate growth with cell fate specification during limb development. Development *135*, 3247–3257.

Thisse, C., and Thisse, B. (2008). High-resolution in situ hybridization to whole-mount zebrafish embryos. Nat. Protoc. *3*, 59–69.

Volckaert, T., and De Langhe, S.P. (2015). Wnt and FGF mediated epithelial-mesenchymal crosstalk during lung development. Dev. Dyn. *244*, 342–366.

Wahl, M.B., Deng, C., Lewandoski, M., and Pourquié, O. (2007). FGF signaling acts upstream of the NOTCH and WNT signaling pathways to control segmentation clock oscillations in mouse somitogenesis. Development *134*, 4033–4041.

Wartlick, O., Mumcu, P., Kicheva, A., Bittig, T., Seum, C., Jülicher, F., and González-Gaitán, M. (2011). Dynamics of Dpp signaling and proliferation control. Science *331*, 1154–1159.

Wendling, O., Dennefeld, C., Chambon, P., and Mark, M. (2000). Retinoid signaling is essential for patterning the endoderm of the third and fourth pharyngeal arches. Development *127*, 1553–1562.

Yu, S.R., Burkhardt, M., Nowak, M., Ries, J., Petrásek, Z., Scholpp, S., Schwille, P., and Brand, M. (2009). Fgf8 morphogen gradient forms by a source-sink mechanism with freely diffusing molecules. Nature *461*, 533–536.

STAR★METHODS

KEY RESOURCES TABLE

REAGENT or RESOURCE	SOURCE	IDENTIFIER
Antibodies		
Chicken monoclonal anti-GFP	Abcam	Cat#13970; RRID:AB_300798
Rabbit monoclonal anti-β-catenin	CST	Cat#8480; RRID:AB_11127855
Mouse monoclonal anti-ppERK	Sigma-Aldrich	Cat#M8159; RRID:AB_477245
Rabbit polyclonal anti-Fibronectin	Sigma-Aldrich	Cat#F3648; RRID:AB_476976
Rabbit polyclonal anti-GFP	ThermoFisher	Cat#A-6455; RRID: AB_221570
Goat anti-Chicken IgY (H+L), Alexa Fluor 488	Invitrogen	Cat#A-11039; RRID:AB_142924
Goat anti-Mouse IgG2b, Alexa Fluor 594	Invitrogen	Cat#A-21145; RRID:AB_2535781
Goat anti-Rabbit IgG (H+L), Alexa Fluor 594	Invitrogen	Cat#A-11012; RRID:AB_141359
Goat anti-Rabbit IgG, Alexa Fluor 647	Invitrogen	Cat#A-21245; RRID:AB_2535813
Anti-Digoxygenin (DIG)-AP Fab fragments	Roche	Cat#1093274; RRID:AB_2314302
Chemicals, Peptides, and Recombinant Proteins		
BCI	CalbioChem	Cat#317496
SU5402	CalbioChem	Cat#572630
BMS493	Sigma-Aldrich	Cat#B6688
Penicillin-Streptomycin	Sigma-Aldrich	Cat#P4333
Antibiotic antimycotic solution (100×)	Sigma-Aldrich	Cat#A5955
L-15 medium with L-Glutamine w/o Phenol Red	GIBCO	Cat#21083-027
Fetal bovine serum (FBS)	ThermoFisher	Cat# A3160601
Fast Red tablets	Roche Diagnostics	Cat# 11496549001
Experimental Models: Organisms/Strains		
Zebrafish: Tg(β-actin:NLS-tdMCP-EGFP)	Campbell et al., 2015	ZFIN: ZDB-TGCONSTRCT-150624-4
Zebrafish: Tg(hsp70l:dnfgfr1a-EGFP)	Lee et al., 2005	ZFIN: ZDB- TGCONSTRCT-070117-101
Zebrafish: Tg(hsp70l:tcf7l1a-GFP)	Lewis et al., 2004	ZFIN: ZDB- TGCONSTRCT-070117-160
Zebrafish: Tg(*hsp70l:dkk1b-GFP*)	Stoick-Cooper et al., 2007	ZDB-TGCONSTRCT-070403-1
Software and Algorithms		
Fiji	Schindelin et al., 2012	https://fiji.sc/; RRID: SCR_002285
MATLAB R2016a	Mathworks	https://www.mathworks.com/products/matlab.html; RRID: SCR_001622
Imaris 8.1.2	Bitplane	http://www.bitplane.com/imaris/imaris; RRID:SCR_007370
GraphPad Prism 7	GraphPad	http://www.graphpad.com/; RRID:SCR_002798
Other		
Nikon A1R GaAsP inverted confocal microscope 40× 1.15 NA apo λS DIC-WI objective	Nikon	N/A
Zeiss Axio-Observer-Z1 ApoTome microscope 40× 0.75 NA dry objective	Zeiss	N/A
Leica DMI 6000 inverted microscope 10× 0.25 NA dry objective	Leica	N/A
Leica M205FA dissection microscope	Leica	N/A

CONTACT FOR REAGENT AND RESOURCE SHARING

Further information and requests for reagents may be directed to the Lead Contact Ertugrul Ozbudak (Ertugrul.Ozbudak@cchmc.org).

EXPERIMENTAL MODEL AND SUBJECT DETAILS

Fish stocks

All the fish experiments were performed under the ethical guidelines of Albert Einstein College of Medicine and Cincinnati Children's Hospital Medical Center, and animal protocols were reviewed and approved by the respective Institutional Animal Care and Use Committees (Protocol # 20150704 and Protocol # 2017-0048). Transgenic (*β-actin:NLS-tdMCP-EGFP*) (Campbell et al., 2015), (*hsp70l:dnfgfr1a-EGFP*) (Lee et al., 2005), (*hsp70l:dkk1b-GFP*) (Stoick-Cooper et al., 2007) and (*hsp70l:tcf7l1a-GFP*) (Lewis et al., 2004), and wild-type adult fish were used in experiments. Adult fish were kept on a 14-10 light/dark cycle at the Zebrafish Core Facility, maintained at 28.5°C. Embryos were collected by natural crosses at the facility and grown in E3 medium until the desired stage. For local heat shock experiments, transgenic heterozygous fish were inbred and wild-type siblings were used as control. Embryos were incubated at 23.5°C for imaging experiments and at 28°C for *in situ* hybridization and immunohistochemistry experiments.

METHOD DETAILS

PSM Tissue Explants

Embryos at different stages (6 – 12 somites) of somitogenesis were dechorionated in E3 medium and deyolked in Leibovitz's cell culture (L-15) medium. Tail bud tissue was dissected and cleaned with microsurgical knife under a stereomicroscope. Needles and lash tools were used to scrape the ventral surface of the tissue to remove the yolk granules.

Imaging slides were prepared with a well to hold the explant in dorsoventral position using two-layers of transparent tape on a 25 × 75 mm microscope slide and cutting out a 15 × 15 mm well in the center that can be coverslipped. Alternatively, double-way slides allowing high-resolution z-stack imaging from either side were prepared with a piece of cardboard cut in the size of a microscope slide with a 20 × 20 mm square hole in the middle. A coverslip (22 × 22 × 0.16-0.19 mm) with two-layer tape thickness well, as described above, was taped to cover the hole. Slide wells were filled with 50 μL of growth medium (L-15 medium with L-Glutamine w/o Phenol Red (GIBCO, 21083-027), %15 FBS (Sigma, F0926), 0.8 mM $CaCl_2$ and 50 U/mL penicillin-streptomycin (Sigma, P4333)).

Embryonic tissue explants were transferred from L-15 medium onto a separate coverslip using glass Pasteur pipettes. The explant was arranged to stay on the coverslip along dorsoventral axis with the help of surface tension after gently sucking out the excess media around it with 20 μL pipettes. This coverslip was flipped over the well-slide filled with growth medium quickly before letting the tissue dry out. Using the influence of physical stress and tension, the explant tissue was maintained with zero axial elongation (non-elongating explant condition) without stalling the somite segmentation (Figure S1).

Imaging and Microscopy

For 3D cell-counting data, multi-dimensional 2 × 4 mosaic images were captured with 5 μm ApoTome z sectioning at 30 minutes intervals on a Zeiss Axio-Observer-Z1 microscope with a 20× dry 0.40 NA objective lens. Mosaic tile images were auto-stitched by using Zeiss AxioVision 4.8 software and cell nuclei were counted as described in "Quantification and Statistical Analysis." Double-way slides were imaged from both dorsal and ventral sides of the embryonic explants, to obtain higher resolution ApoTome sections beyond 40-50 μm of tissue depth at each time-point. Microscope room was kept temperature controlled at 23°C throughout the experiments.

Data for the elongating/non-elongating explant conditions and drug-treatment experiments were taken on a Leica DMI 6000 inverted microscope with 10× 0.25 NA dry objective lens and DFC-340-FX camera at 25°C. Transmission light and GFP fluorescence images were taken every 3 minutes over 6 hours for the segmentation period measurements. Scaling experiments were performed by taking hourly images for 8 hours.

NBT/BCIP stained *in situ* hybridization samples were imaged on a Leica M205FA dissection microscope. Samples of *xirp2* staining were imaged in Petri dishes covered with agarose as whole embryos (wedged-shaped troughs made with a plastic mold plate in 1.5% agarose/maleic acid buffer (MAB) solution). Samples of *cyp26a* staining were flat mounted on a coverslip-slide chamber using nail polish before imaging. Fast Red stained fluorescent *in situ* hybridization samples were imaged with 2 μm z-scan on a Nikon A1R GaAsP inverted confocal microscope with a 20× apochromatic VC DIC 0.75 NA dry objective lens. Maximum intensity projection of five consecutive z-steps (10 μm) were used to analyze fluorescence intensity.

Fixed samples in immunostaining protocols (WT, heat shocked, or transplanted embryos and dissected, or local heat shocked explants) were imaged in slide nail polish chambers (lateral for late, flat mounted for early stages). Either a Zeiss Axio-Observer-Z1 microscope with a 40× dry 0.75 NA (1 μm ApoTome z sectioning) or a Nikon A1R GaAsP inverted confocal microscope with a 40× apochromatic λS DIC-water immersion 1.15 NA objective lens (2 μm sectioning) were used for imaging.

Pharmacological Treatments

To perturb the determination front regression dynamics during somitogenesis, embryos were treated either with SU5402 (CalbioChem, 572630; 2 – 20 μM) FGF receptor inhibitor drug (Sawada et al., 2001) or BCI (CalbioChem, 317496; 0.5 – 10 μM) to inhibit dual-specificity phosphatase (DUSP1/6) downstream auto-inhibitor of FGF signaling (Molina et al., 2009). 10 μM SU5402

treatment was performed at 28°C for predetermined somites counting experiments. RA signaling was inhibited with BMS493 (Sigma, B6688; 50 μM) retinoic acid receptor inhibitor drug (Wendling et al., 2000). Working solutions of drugs (1 mM for SU5402, 10 mM for BCI and 10 mM for BMS493) were prepared in DMSO and diluted in either E3 medium (for whole embryos) or growth medium (for explants) to their final concentrations. Control data were obtained with corresponding concentrations of DMSO. The final concentration used in each experiment is provided in the legends of each figure.

Heat shock Experiments

12 somite stage *hsp70l:tcf7l1a-GFP* or *hsp70l:dnfgfr1a-EGFP* and 11 somite stage *hsp70l:dkk1b-GFP* transgenic embryos were heat shocked at 38°C for 50 or 60 minutes, respectively. Embryos were either fixed immediately after the heat shock or with one hour delay for immunostaining. For segment size changes due to Wnt/FGF inhibition, embryos were grown at 28°C incubator following the heat shock. For transplantation experiments, same duration (50 minutes) heat shock is applied to both donor and host embryos, at 8-12 somite stages, grown overnight at 23°C in transplantation media following transplantation protocol.

Local Heat shock Experiments

8 somite stage embryos were dissected into anterior (anterior somites) and posterior (2-3 posterior somites and the PSM) pieces. Both tissue pieces were flat mounted in imaging slide wells as described above. The anterior tissues were heat shocked for 35 minutes in water bath at 38°C to identify transgenic embryos. The posterior tissues were locally heat shocked using the UV light (405 nm) exposure in 63× oil objective (NA = 1.25) with a Leica DMI-6000 inverted microscope. Exposure area was restricted by using one of three different pinholes (circular and mediolateral or rostrocaudal alignment of rectangular). The duration and power of UV exposure were optimized as 400 s and 55% fluorescence diaphragm at full power of Leica EL6000 UV light source. Transgenic identified tissues were analyzed whereas wild-type siblings were kept as control.

Cell Transplantation

Transplantation media (1:100 Antibiotic Antimycotic Solution (100×, Sigma, A5955) diluted in 1/3 Ringer solution) was prepared fresh before the experiments. 25 mm plastic Petri dishes were covered with 2% agarose in transplantation media for the procedure and holding dechorionated young embryos. Agarose solution in the transplantation dish was molded with diagonal holes sitting one embryo in each. All steps were performed embryos soaked in the transplantation media. Wild-type host and transgenic donor embryos were dechorionated manually in glass dishes starting 2 hpf. Host embryos were maintained slightly younger than the donor embryos. When donor cells got small enough to fit into transplantation needle, 40-50 cells were transplanted from donor to host embryos. In order to increase mesoderm fate efficiency of transplanted cells, cells were collected from the blastoderm near yolk margin of donor embryos and targeted into similar latitudes of host embryos.

In Situ Hybridization

Embryos were stained for *xirp2*, *cyp26a1* or *fgf8* expression. DIG labeled RNA probes were prepared by *in vitro* transcription and anti-digoxygenin (DIG)-AP Fab fragments (Roche, 1093274) were used. Embryos raised at 28°C were fixed (30 hpf for *xirp2* experiments, at indicated somite stages for *cyp26a1* and *fgf8* experiments) with 4% paraformaldehyde (PFA) at room temperature for 2 hours. Fixed embryos were washed in MAB-Tween, dehydrated in methanol and rehydrated following previously reported standard *in situ* hybridization protocols (Thisse and Thisse, 2008). *fgf8* staining is colored with fast red tablets for fluorescent imaging and quantification.

Immunohistochemistry

For ppERK and β-catenin immunostaining, embryos were collected at 10 different stages (3, 4, 5, 10, 11, 12, 14, 18, 22 and 26 somites), dechorionated with needles, anesthetized with tricaine, fixed overnight at 4°C with 4% PFA and dehydrated with methanol. Explants were fixed following flat mounting in slide chambers. After being rehydrated, samples were washed with MAB-D-T (150 mM NaCl in 0.1 M maleic acid buffer, pH 7.5, 1%DMSO and 0.1% Triton X-100 detergent) and blocked with fetal bovine serum (FBS) in MAB-D-T for 2 hours at room temperature. Samples were then incubated with monoclonal mouse antibody against pp-ERK (1:2000, Sigma) and monoclonal rabbit antibody against β-catenin (1:200, Cell Signaling) in MAB-D-T at 4°C for 24 hours, washed with MAB-D-T and incubated overnight in Alexa Fluor 597 goat anti-mouse IgG2b (1:200, Invitrogen) and Alexa Fluor 647 goat anti-rabbit IgG (1:200, Invitrogen) secondary antibodies in MAB-D-T. After several MAB-D-T washes and Hoescht 33342 staining of cell nuclei (1:5000), embryos were imaged.

For local heat shock experiments, tissues were fixed 10 hours after the experiments at 4°C with 4% PFA overnight and dehydrated with methanol. Following rehydration, tissues were membrane permeabilized with 2% PBS-T (phosphate buffer saline and 2% Triton X-100 detergent) for 1 hour and blocked with normal sheep serum in PBS-Tw-B (PBS, 0.1% Tween-20 detergent and 2% bovine serum albumin) for 2 hours at room temperature. Tissues were then incubated with polyclonal rabbit antibody against GFP (1:100, ThermoFisher) in PBS-Tw-B at 4°C overnight, washed with PBS-Tw and incubated 4 hours in secondary Alexa Fluor 594 goat anti-rabbit IgG (H+L) (1:200, Invitrogen) antibody and Hoescht33342 (1:4000) in PBS-Tw-B. After several PBS-Tw washes, tissues were imaged.

For transplantation experiments, embryos were fixed 6-8 hours after the 50 minutes heat shock. Monoclonal chicken anti-GFP (Abcam, 13970, 1:400), mouse anti-pp-ERK (1:1000) and rabbit anti-Fibronectin (Sigma, F3648, 1:200) antibodies were used as primary. Alexa fluorophore conjugated secondary antibodies (488, 568 and 647 respectively for each species, 1:200) were used in combination of Hoescht 33342 (1:5000) nuclear staining at room temperature. Images from host embryos were analyzed subsequently whereas donor embryos were kept as control for efficiency of heat shock and immunostaining.

Counting Predetermined Somites

20-30 embryos were picked for every other somite stage, from 2 somite stage up to 28 somite stage, to quantify number of predetermined somites through somitogenesis. Embryos were treated with SU5402, FGFR inhibitor drug, making FGF gradient shallower and resulting determination of larger somites. The drug was washed out after 50 minutes and embryos were raised till the end of somitogenesis (30 hpf) before being fixed for visualizing somite boundaries with *xirp2 in situ* hybridization.

As anterior PSM cells were already predetermined to segment into somites, a fixed number of somites segmented with normal sizes (predetermined somites) prior to formation of larger somites due to the immediate effect of drug on FGF gradient. We calculated the number of predetermined somites at each stage of somitogenesis by subtracting the somite number at the beginning of drug treatment from the number of first large somite formed due to drug treatment (Figure S1G).

QUANTIFICATION AND STATISTICAL ANALYSIS

Signaling Network Model

The posterior signaling network (FGF or Wnt) is built consisting of the following network elements: Ligand mRNA (mLIG), Diffusible Ligand Protein (LIG), Ligand-Receptor-Complex (COMP), inactive signal protein (SIG^-), active signal protein (SIG^+), and inhibitor protein (INH), as depicted in Figure 3A. We assumed sufficient levels of receptors were present in the system for ligand binding. Using synthesis (transcription for mRNA or translation for protein), degradation, ligand-receptor binding, signal activation, and signal deactivation steps, the rates of change of the concentration of network elements for the kth cell in the PSM at any given time t are given with the following rate equations:

$$\frac{\partial(mLIG_k)}{\partial t} = k_{mLIG}^{syn} - mLIG_k \times k_{mLIG}^{deg}$$

$$\frac{\partial(LIG_k)}{\partial t} = (mLIG_k)\,|_{t-trlatedel} \times k_{LIG}^{syn} - LIG_k \times k_{LIG}^{deg} - (LIG_k)\,|_{t-actdel} \times k_{LIG}^{bin} + D \times \frac{\partial^2(LIG_k)}{\partial x^2}$$

$$\frac{\partial(COMP_k)}{\partial t} = (LIG_k)\,|_{t-actdel} \times k_{LIG}^{bin} - COMP_k \times k_{COMP}^{deg}$$

$$\frac{\partial\left(SIG_k^-\right)}{\partial t} = k_{SIG}^{syn} + SIG_k^+ \times INH_k \times k_{SIG}^{deact} - SIG_k^- \times COMP_k \times k_{SIG}^{act} - SIG_k^- \times k_{SIG}^{deg}$$

$$\frac{\partial\left(SIG_k^+\right)}{\partial t} = SIG_k^- \times COMP_k \times k_{SIG}^{act} - SIG_k^+ \times INH_k \times k_{SIG}^{deact} - SIG_k^+ \times k_{SIG}^{deg}$$

$$\frac{\partial(INH_k)}{\partial t} = SIG_k^+\,\Big|_{t-reprdel} \times k_{INH}^{syn} - INH_k \times k_{INH}^{deg}$$

Here we introduced time delays for three cellular processes: *trlatedel* for translation of LIG, *actdel* for activation cascade downstream of COMP, and *reprdel* for transcriptional activation, transcription, and translation of INH. We defined four synthesis (transcription of mLIG, k_{mLIG}^{syn}; translation of LIG, k_{LIG}^{syn}; transcription and translation of SIG, k_{SIG}^{syn}; translation of INH, k_{INH}^{syn}) and five degradation (degradation of mLIG, k_{mLIG}^{deg}; degradation of LIG, k_{LIG}^{deg}; degradation of COMP, k_{COMP}^{deg}; degradation of SIG, k_{SIG}^{deg}; degradation of INH, k_{INH}^{deg}) rates for dynamic equations. These twelve rate parameters of the system are set constant to biologically relevant values (Table S1) so that the shape of the mRNA gradient is approximated to in situ hybridization pictures available. We assumed diffusion of ligands obeys Fick's Law, i.e. one-dimensional diffusion speed (D) is constant throughout the PSM independent of concentrations. k_{LIG}^{bin} stands for receptor-ligand binding rate. k_{SIG}^{act} and k_{SIG}^{deact} are signal protein activation and deactivation rates respectively. These four parameters are kept as variable knobs in the simulations.

Simulations

We performed discrete time evolution solutions for these equations at a simulation area represented with one-dimensional matrix of posteroanterior 85 cells. Only posteriormost 15 cells were allowed to transcribe ligand mRNA (mLIG) throughout the simulations, with k_{mLIG}^{syn} rate (first equation). The model is simulated for 31 segmentation cycles in total: (1) We first simulated formation of steady posterior gradient for 14 segmentation cycles in normal elongation conditions. To represent normal posterior elongation of the PSM we added a new cell to the tail bud after every one fifth of segmentation cycle starting with same matrix values of its anterior neighbor. We kept the simulation area unchanged by deleting the anteriormost cell as a new posterior cell is added to the system. (2) We next simulated non-elongating condition (no addition of new cells from the posterior end) for 17 additional segmentation cycles.

Out of 16 reaction parameters, we varied the ligand diffusion speed (D), ligand receptor binding rate (k_{LIG}^{bin}), and SIG activation (k_{SIG}^{act}) and deactivation (k_{SIG}^{deact}) rates over parameter windows of 10 equally spaced values (10,000 parameter sets). Parameter windows are determined to allow a variety of gradient shapes in normal elongation simulations from very steep to very shallow, i.e. leg of the slope sweeping more than half of the PSM cells. The ligand diffusion speed (D) is set to zero for the no-diffusion model (Figure 3B).

One segmentation cycle was set as 70 simulation time steps and the equations were evolved in 100 iterations for recording FGF network elements matrix columns corresponding to every simulation time step. Concentrations of six elements of the network (mLIG, LIG, COMP, SIG^-, SIG^+, INH) are calculated for each time step and logged in 2-D matrices of $85(L)\times2170(T)$ dimensions. All matrices started from zero at t=0. Diffusion was allowed only anteriorly from the posteriormost cell (no-flux boundary condition) and there was no diffusion posteriorly from the anteriormost boundary cell (perfectly absorbing boundary condition). We can rewrite the diffusion term in the second equation for 1D discrete matrix taking into consideration these boundary conditions as:

$$\frac{\partial^2(LIG_k)}{\partial x^2}=\begin{cases}\dfrac{LIG_{k=2}-LIG_{k=1}}{\Delta x^2} & k=1\\[2mm] \dfrac{LIG_{k+1}+LIG_{k-1}-2LIG_k}{\Delta x^2} & 1<k<85\\[2mm] 0 & k=85\end{cases}$$

SIG^+ matrices were interpolated linearly to micron scale by giving 8 μm size to every cell (i.e. five cells corresponding to regular somite size, 40 μm) before calculating the determination front readouts as described in the following section.

The sizes of newly determined somites were calculated as the positional shift of the determination front during each clock cycle. Simulated scaling data were then plotted as sizes of somites vs. sizes of PSM at segmental determination, calculating the PSM size at segmental determination by adding sizes of predetermined somites to the determination front position.

Tail bud dissection experiments were simulated as the removal of posterior-most 15 cells from the simulation window after formation of a steady gradient. We assigned a decreased k_{SIG}^{act} rate after the FGFR inhibitor drug, SU5402, treatment. We assigned a decreased k_{SIG}^{deact} rate after the DUSP1/6 inhibitor drug, BCI, treatment. When whole embryos were treated with low SU5402 concentration (2 μM) continuously, the peak in somite size was reached after two segmentation cycles (Figure 6E). Hence, we assumed the drug acting slowly by gradually decreasing k_{SIG}^{act} rate over a period of two clock cycles.

For whole embryo elongation simulations: we measured the tail bud displacement by adding the displacement of somite boundary (size of the last formed somite) to the change of the PSM size (Figure S1E). As the number of predetermined somites decrease during somitogenesis from five to one (Figure S1G), after tail bud stage, the determination front specifies sizes of 25 somites in 25 clock cycles while 30 somites form. Hence, we resampled average elongation speeds for every segmentation cycle from tail bud displacement data (Figure S1F); this information was used as input in simulations.

For mosaic heat shock simulations, same parameter set used in scaling explant simulations was used and the heat shock is applied to 20th, 21st and 22nd cells from posterior in a 75 cell-length PSM. Effect of the heat shock is simulated as drop of ERK phosphorylation rate for 14 simulation time steps within the segmentation cycle #0. The position of the determination front was between 30th and 31st cell at the beginning of cycle #0. Cells determined to group into a somite at the end of each cycle (Figure 7F) for 6 cycles (T-2 to T+3) as the PSM was allowed to grow posteriorly with constant speed (5 cells/segmentation cycle) are indicated.

The simulation codes have been implemented in MATLAB 2015b and 2016a. The current version of our code can perform a 31 segmentation cycle simulation of 85 PSM cells in less than 1 minute (on Windows10, with 2.30 GHz Intel Core i5 and 8 GB of RAM).

Determination Front Readouts from Simulations

Determination front was initiated from the experimentally determined position at a specific somite stage. For regression of the determination front, readout (R) matrices were calculated from the SIG^+ matrix, per descriptions in Figure 3C, as follows:

i-) For "constant threshold" determination front was shifted at the end of every clock cycle to read the same threshold value as fixed at t=0:

$$SIG^+\big|_{t}^{df_1}=R=SIG^+\big|_{t-T}^{df_0}$$

where R is the readout at the determination front, T is the segmentation period, df_1 and df_0 are the positions of determination front at that moment (t) and one cycle earlier respectively.

ii-) For "temporal integration" the value of SIG+ matrix columns over one clock cycle (70 simulation time steps) were summed for each positional row and the position of determination front was set at the end of every clock cycle to read the same sum value as at the initial position at t=0:

$$\sum_{t-T}^{t} SIG^{+}\Big|^{df_1} = R = \sum_{t-2T}^{t-T} SIG^{+}\Big|^{df_0}.$$

iii-) For "time derivative" the value of SIG+ matrix element at any given moment was subtracted by the value read 10 simulation time steps (t_0) earlier and then normalized by the original value in any given positional row. Determination front regressed to its new position at the end of every clock cycle to read the same readout matrix value:

$$\frac{SIG^{+}|_{t-t_0}^{df_1} - SIG^{+}|_{t}^{df_1}}{SIG^{+}|_{t}^{df_1}} = R = \frac{SIG^{+}|_{t-T-t_0}^{df_0} - SIG^{+}|_{t-T}^{df_0}}{SIG^{+}|_{t-T}^{df_0}}.$$

iv-) For "spatial derivative" the difference between the SIG+ matrix values read at the determination front position at t=0 and at the last segment's anterior border (40 μm away) was calculated for t=0. At the end of every clock cycle, the place giving same differential reading with previous determination front position was then defined as the new position of determination front:

$$\frac{SIG^{+}|_{t}^{df_1} - SIG^{+}|_{t}^{df_0}}{df_1 - df_0} = R = \frac{SIG^{+}|_{t-T}^{df_0} - SIG^{+}|_{t-T}^{df_{-1}}}{df_0 - df_{-1}}.$$

v-) For "spatial fold change" the fractional difference between the SIG+ matrix reading of every positional row and its posterior neighbor cell (8 μm away) was calculated as the readout matrix. We summed the SIG+ matrix elements over 8 μm windows (D) centered on the position of interest as one cell data. The determination front then regressed to its new position at the end of every clock cycle to maintain the constant readout:

$$\frac{\sum_{df_1+D/2}^{df_1+3D/2} SIG^{+}|_{t} - \sum_{df_1-D/2}^{df_1+D/2} SIG^{+}|_{t}}{\sum_{df_1-D/2}^{df_1+D/2} SIG^{+}|_{t}} = R = \frac{\sum_{df_0+D/2}^{df_0+3D/2} SIG^{+}|_{t-T} - \sum_{df_0-D/2}^{df_0+D/2} SIG^{+}|_{t-T}}{\sum_{df_0-D/2}^{df_0+D/2} SIG^{+}|_{t-T}}.$$

Calculation of PSM Size at Segmental Determination

The PSM size at segmental determination of the kth somite $(sdPSM_k)$ in the non-elongating explants was calculated by adding the sizes of predetermined somites (S) at segmental determination of the kth somite to the size of the PSM when k^{th} somite is forming as:

$$sdPSM_k = PSM_k + \sum_{j=k-npds}^{k-1} S_j$$

where *npds* is the number of predetermined somites (Figure S2A).

The PSM size at segmental determination of the kth somite in intact zebrafish embryos was equal to the size of the PSM (Figure S2) when the kth somite was predetermined:

$$sdPSM_k = PSM_{k-npds}$$

For chicken and mouse, we used the anterior limit of *Mesogenin* expression domain (*MSGN*) as a proxy for the determination front position (Gomez et al., 2008). Therefore, the PSM size at segmental determination of the kth somite in the chicken and mouse embryos was calculated as:

$$sdPSM_k = PSM_{k'} \text{ for such earlier } k' \text{ giving } PSM_{k'} - MSGN_{k'} = \sum_{j=k'+1}^{k} S_j.$$

Image Analysis

3D Cell Counting Experiments

Dorsal and ventral z-stacks of 3D cell counting data were matched post-acquisition by tracing the constellating bright nuclei and overlapping after upside-down image reflection in Fiji.

Non-PSM (the notochord, adaxial and skin) tissues were trimmed out, contrast and brightness of 16-bit grayscale images were corrected, *subtract background*, *despeckle* and *ROF denoise* functions were used in Fiji to optimize the image. Images were reconstructed using the *gray morphology* tool and the *find maxima* tool was used to count the cell nuclei.

We excluded data from the curved tail bud region of the PSM ($L_{tailbud} = 110 \pm 10\ \mu m$ and $N_{tailbud} = 980 \pm 30\ cells$) for conversions between 3D cell counting and 1D length measurement plots (Figure 1F). Remaining volume of the PSM was modeled as a bilateral prism with equal lengths, $L_{PSM} - L_{tailbud}$, and cross section areas, A. The somites were shaped as closed quadric volumes, with principal cross section area A and orthogonal (anteroposterior) diameter L_S. Hence, the volumes of tail bud-excluded PSM and somite were respectively calculated as:

$$\frac{(N_{PSM} - N_{tailbud})V_{cell}}{\varphi_{sc}} = 2A(L_{PSM} - L_{tailbud})$$

and

$$\frac{(N_S)V_{cell}}{\varphi_{fcc}} = \frac{2}{3}AL_s$$

where V_{cell} is the average volume of a single cell. Here we assumed a simple cubic arrangement for the PSM ($\varphi_{sc} \approx 0.52$) and a face-centered cubic arrangement for the somites ($\varphi_{fcc} \approx 0.74$). Dividing these two formulae side by side:

$$\frac{3\varphi_{sc}}{\varphi_{fcc}} \frac{N_S}{(N_{PSM} - N_{tailbud})} = \frac{L_S}{(L_{PSM} - L_{tailbud})}$$

gave us a correction coefficient from 3D to 1D scaling plots as $3\varphi_{sc}/\varphi_{fcc} \approx 2.108$.

Quantification of Immunohistochemistry Data

ppERK and β-catenin^{nuclear} staining intensity were measured in Fiji in rectangular ROIs centered in the PSM abstaining from the signal coming outside of PSM, i.e. skin, adaxial and notochord cells. Curved PSM images from late somite stages were straightened using warp tool of Adobe Photoshop CC 2015.5 without altering the surface area of images before plotting rectangular intensity profiles in Fiji. For nuclear localized signal, Hoescht 33342 staining of cell nuclei were used to create an image mask for data. Average intensity profiles per cell nuclei through PSM axis were than calculated by dividing the masked image profile by the profile of binary mask itself. Intensity profiles from each embryo were normalized between minimum and maximum PSM signals, and overlaid by matching anterior ends of the PSM. Normalized intensity plots were averaged out between all embryos at each stage / condition and smoothed with GraphPad Prism 7 software using sixth order polynomial and data from hundred neighboring pixels. The percentage of signal output (ppERK and β-catenin^{nuclear}) intensity difference between neighboring cells, for spatial fold change model, was calculated by using a cell size of 6.8 μm (average distance measured between neighboring PSM cell nuclei at Fiji by analyzing nuclear localized fluorescence signal). The slope of signal output, for spatial derivative model, was calculated using known sizes of the last predetermined somite for each stage (Figure S1). All readout calculations from immunohistochemistry used the same formulae used for simulations. Data from different stages / conditions is presented as posterior ends of the PSM (tail bud) overlaid except Figures S5D and S5E. In Figures S5D and S5E data is overlaid from anterior (formed somites) to indicate stepwise regression of the ppERK profile between consecutive somites.

Quantification of Transplantation Data

Transplantation images from confocal microscopy were analyzed in Imaris 9.0.1 software. Nuclear staining was used to identify surfaces for each cell. Median intensity values above a certain threshold from green and red channels were used to identify GFP+ cells and ppERK expressing posterior PSM surfaces, respectively. Nearest neighbor calculations for cells were performed applying distance formula (d<10 μm) to 3D position data of identified surfaces. Fibronectin images were used to draw borders of precocious somites formed due to transgenic cells in the PSM.

Statistical Analysis

Experimental scaling data were averaged out for every new somite forming, after the predetermined ones, and plotted with their standard deviations. All n numbers indicate the number of embryos and/or PSM tissue explants. Mean and error (s.d., s.e.m. and/or confidence interval as indicated) calculations of data are done in GraphPad Prism software.

Simulation scaling trends for different parameter sets were compared with experimental data in logarithmic scale with ANCOVA (built-in *aoctool* function in MATLAB). Both the slope and y-intercept of linear fits in log-log scale were required to pass a significance threshold of $\alpha < 0.2$ with built-in *multcomp* statistical comparison function for a successful parameter set.

For all statistical significance results in between intensity quantifications from immunostaining data and consecutive calculations, we used non-parametric Student's t test (without Gaussian distribution assumptions), with Kolmogorov-Smirnov method.

DATA AND SOFTWARE AVAILABILITY

The collection of MATLAB scripts used to run the simulations and fit the experimental data with simulations are available online (https://github.com/mfsimsek/scalingpaper).

Cell Reports, Volume *24*

Supplemental Information

Spatial Fold Change of FGF Signaling Encodes Positional Information for Segmental Determination in Zebrafish

M. Fethullah Simsek and Ertuğrul M. Özbudak

SUPPLEMENTARY FIGURES AND TABLE

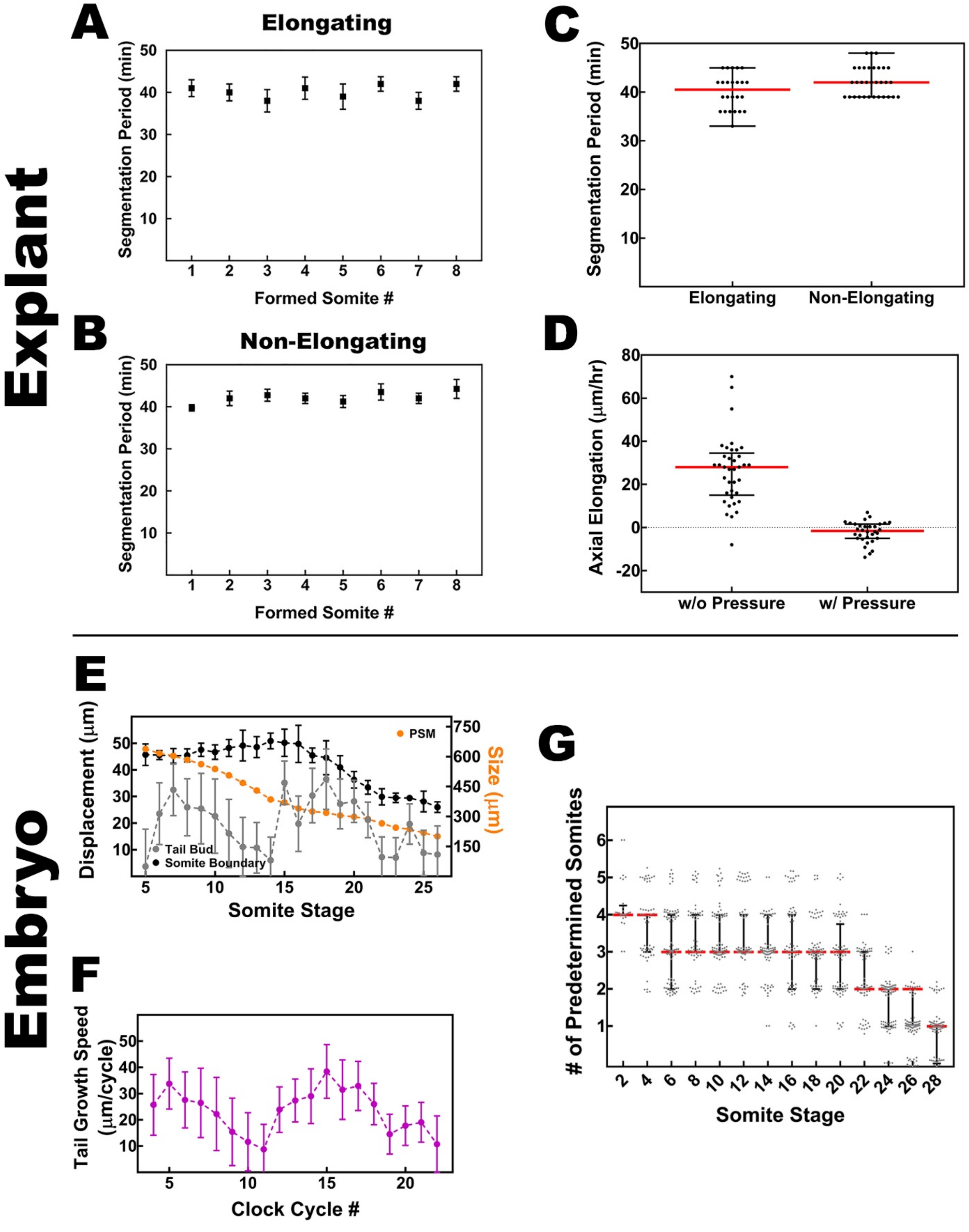

Figure S1. Characterization of 3-D PSM Explants and Quantification of Somitogenesis Dynamics in Intact Embryos, Related to Figures 1, 2, 4, 6, and 8

(A,B) Segmentation period does not change in PSM tissue culture during formation of consecutive somites under elongating (N=4) or non-elongating (N=5) conditions. Error bars indicate s.e.m. (C) Average period also doesn't change in between elongating and non-elongating conditions. Red lines show medians of data. (D) Average axial elongation speeds of explants measured over six hours in culture. Explants stops their axial elongation under pressure (error bars s.d.). (E) Size of presomitic mesoderm (orange, right axis) measured through mid-somitogenesis, from 5 somite stage until 25 somite stage. Displacement of tail bud (gray) in between formation of each somite is calculated from the sum of the change in PSM size and displacement of last somite boundary (size of last formed somite, black). (N=6-20, mean with s.d.). (F) Average axial elongation speed per clock cycle (purple, with s.d.) is calculated from resampling of tail bud displacement from somite stage into segmentation clock cycles using change of predetermined somites data (G). (G) Number of predetermined somites measured, from 2 somite stage until 30 somite stage (N>27). Median values are shown with red lines. Error bars represent quartiles of the data. Original data (integers) are jittered with 0.05 standard deviation keeping mean matching data within 0.01 range, to make data visible (gray dots).

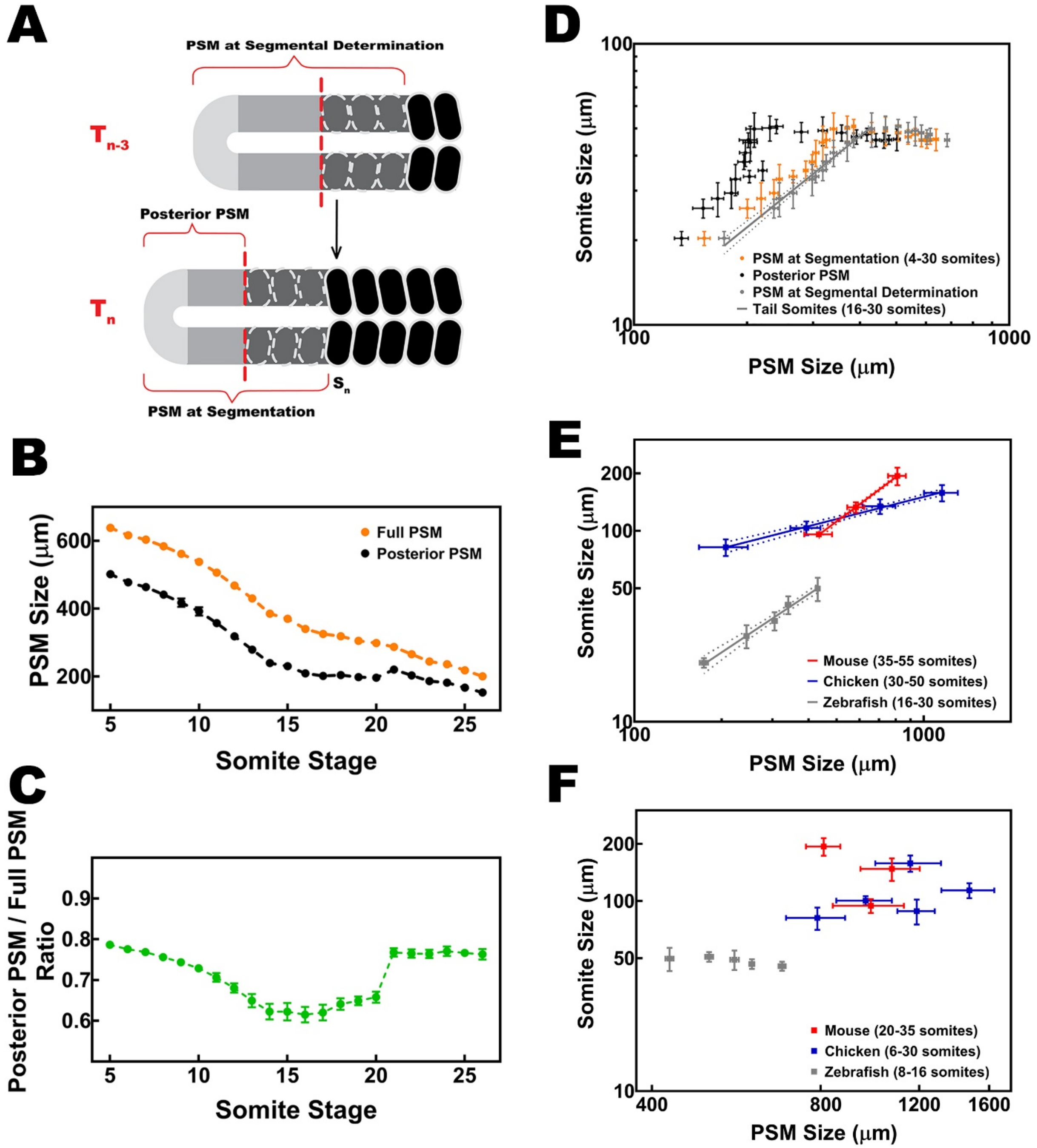

Figure S2. Scaling between Sizes of Tail Somites and the PSM Tissue, Related to Figures 1, and 4

(A) Sketch of PSM and last formed somites at a stage, S_n, when there are three predetermined somites in the anterior PSM (bottom) and the PSM and last formed somites when that n^{th} somite was determined (top). (B) Size measurements for these tissues through somitogenesis. (C) The ratio of posterior PSM versus full PSM is not kept constant. The relative position of the determination front nonlinearly changes in the PSM. (D) The sizes of tail somites scales only with the size of PSM at segmental determination but not with the size of PSM at segmentation or posterior PSM. The anterior somites do not display scaling. (E) Tail somites are segmented in gradually decreasing sizes scaling in size with the PSM at segmental determination for three classes of vertebrate species (reanalyzed from Gomez et al. 2007). (F) The anterior somites do not display scaling in three species. Error bars indicate s.d.

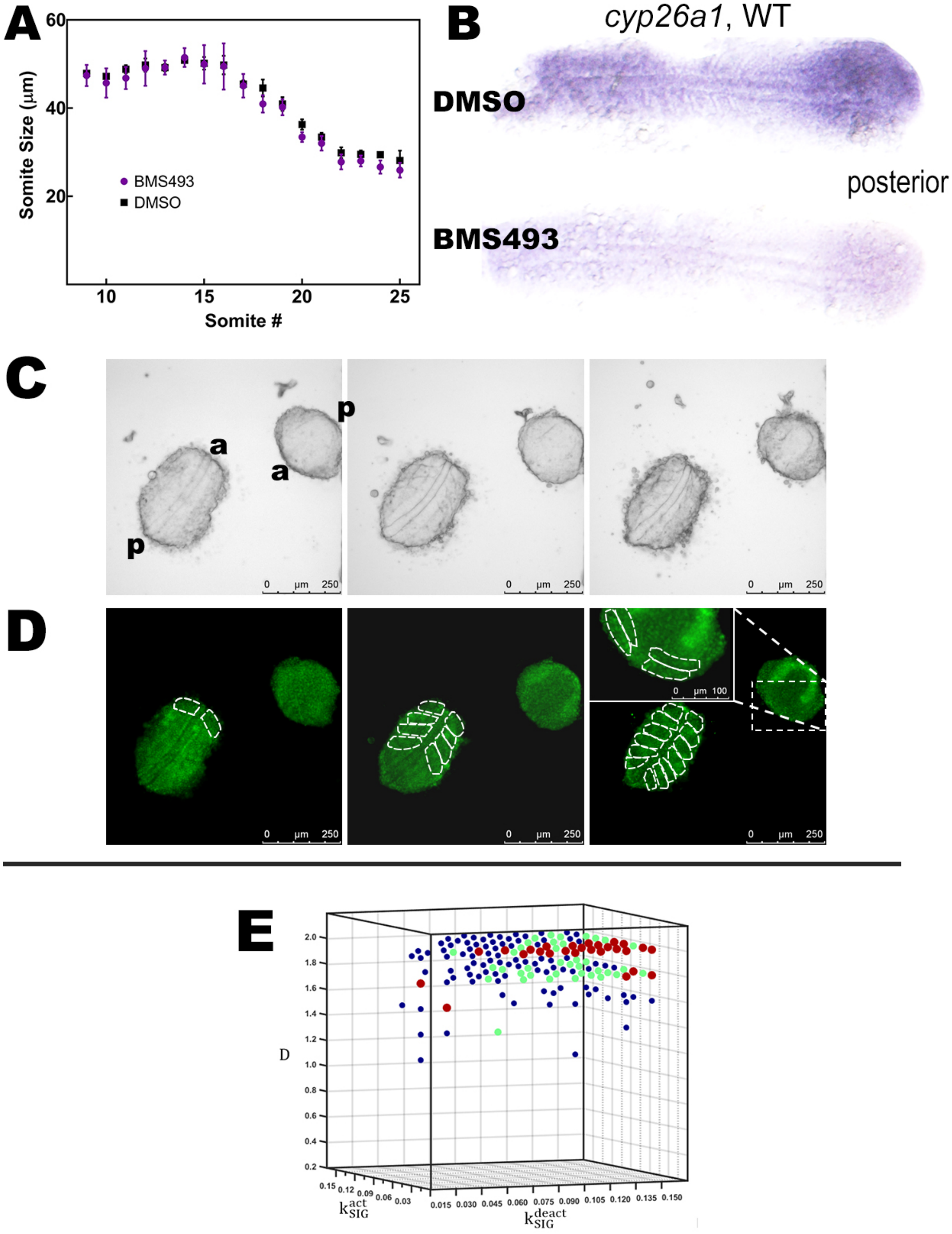

Figure S3. RA Gradient is Inhibited with BMS493 Treatment and Half PSM Explants Wait for Anterior Somites before Segmentation, and Simulations Parameter Space **Related to Figures 2, 3, and 6**

(A) Whole embryos continuously treated with pan-RAR inhibitor BMS493 drug (50 µM for 7 hours, starting at 10 somite stage, N=7) or with DMSO only (1%, corresponding concentration, N=15) formed somite segments with similar sizes. Error bars s.e.m. (B) Transcription of *cyp26a1* is reduced in embryos treated with same concentration of BMS493 for 1 hour compared to DMSO treated embryos. (C) Bright field images of anterior and posterior pieces of the PSM explant without any somites at t=0 (left), t=2 hours (middle) and t=5 hours (right). Short posterior piece of the PSM explant is placed away from and behind the anterior end of the anterior piece and flipped in opposite anteroposterior orientation, to ascertain segmentation is not altered by any kind of morphogen diffusion in between tissues. (D) Images of same explants in green channel showing signal from nuclear expressed GFP marker. Starting from S0 at t=0, borders of forming somites are outlined as dashed white lines. (E) Spread of 243 parameter sets (out of 10,000 tested) giving successful (passing statistical criteria as described in Methods) fit for normal scaling data from PSM explants under non-elongating conditions. X, Y, and Z axes are signal protein deactivation (k_{SIG}^{deact}) and activation (k_{SIG}^{act}) rates and ligand diffusion speed (D) respectively. Different ligand receptor binding rates (k_{LIG}^{bin}), are shown in different colors, size of markers increase as the binding gets stronger. Only three lowest out of ten binding rates gave successful results within the parameter space.

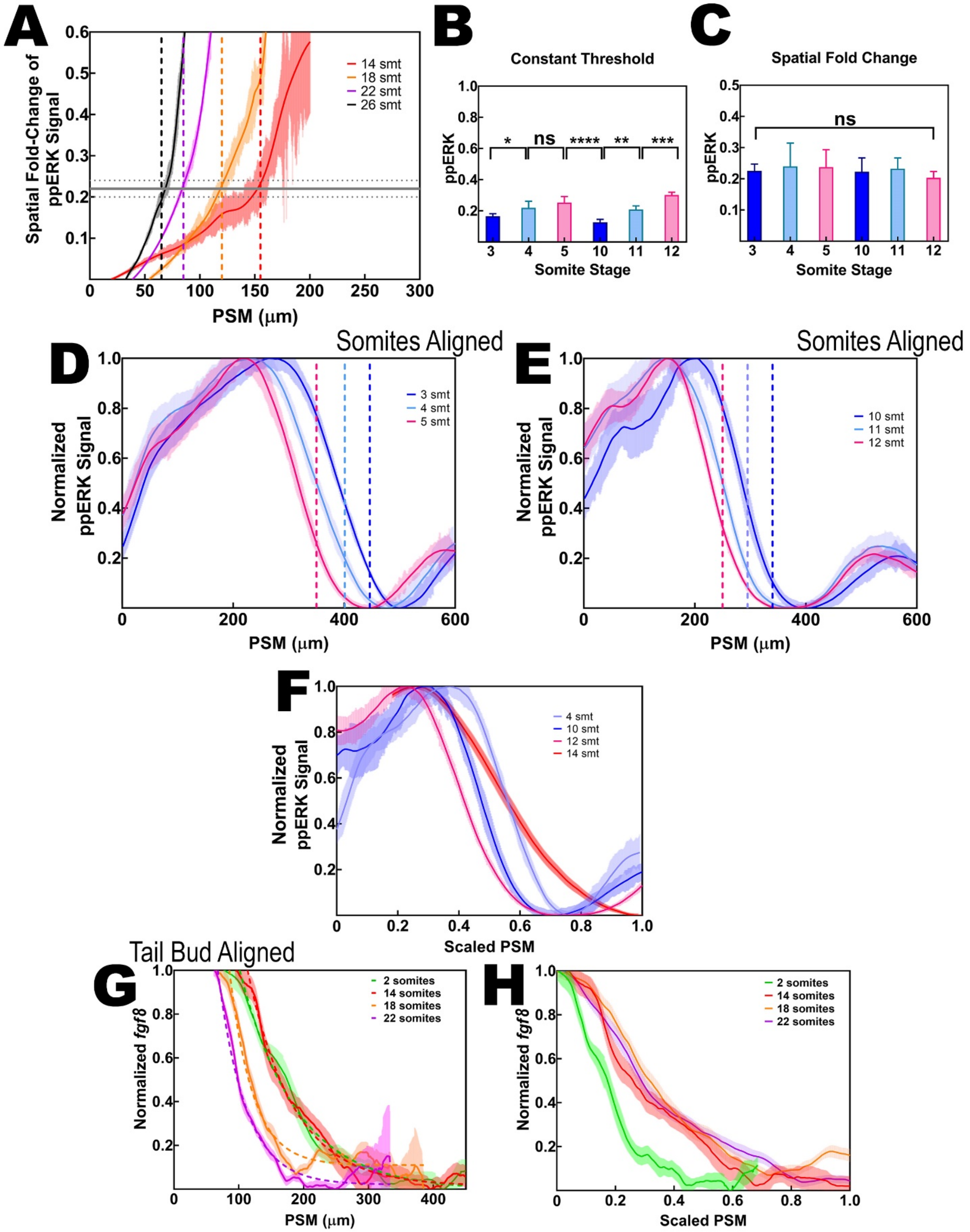

Figure S4. Spatial Fold Change of FGF Signaling at Different Stages and Both *fgf8* mRNA and ppERK Gradients Scale with the PSM size at Late but not Early Stages, Related to Figures 4, and 5

(A) The SFC readout of ppERK levels (n = 17, 9, 14 and 13 for 14, 18, 22 and 26 somite stages, respectively). Posterior end of the PSM are matched for all stages. Determined-undetermined PSM borders are marked by dashed vertical lines with stage matching colors. At the determination front (vertical dashed lines), the SFC of ppERK reads out precisely the same level (22 ± 2%, gray horizontal line) at different stage embryos. (B, C) The SFC (C) but not absolute level (B) of ppERK is conserved at the determination front at 3, 4, 5, 10, 11 and 12 somite staged embryos. Error bars indicate s.d. Note that the absolute level of ppERK is only conserved at stages 4 and 5 somite stages due to the fact that PSM size do not change appreciably at those stages (Gomez et al., 2008), which led to the wrong conclusion that a constant level of ppERK moves stepwise during segmentation (Akiyama et al., 2014). (D, E) Normalized levels of ppERK (n = 20, 14, 16, 14, 12, and 14 for 3, 4, 5, 10, 11, and 12 somite stages) quantified as FGF signaling output. Data is aligned from anterior (formed somites) within each group of consecutive stages. Determined-undetermined PSM borders are marked by dashed vertical lines with stage matching colors, respectively. ppERK profile shows stepwise regression in between consecutive stages determining similar sized somites. (F) Unlike later stages (Figure 4D), ppERK gradient at early stages do not scale with the size of the tissue between posterior tip of notochord and anterior end of the PSM. (G) Normalized levels of *fgf8* mRNA gradient (n = 14, 19, 18, and 18 for 2, 14, 18, and 22 somite stages) quantified as FGF signaling input. (H) *fgf8* mRNA gradient scale with the size of PSM at 14, 18 and 22 somite stages but not at 2 somite stage. Shaded regions are s.e.m. Posterior is left.

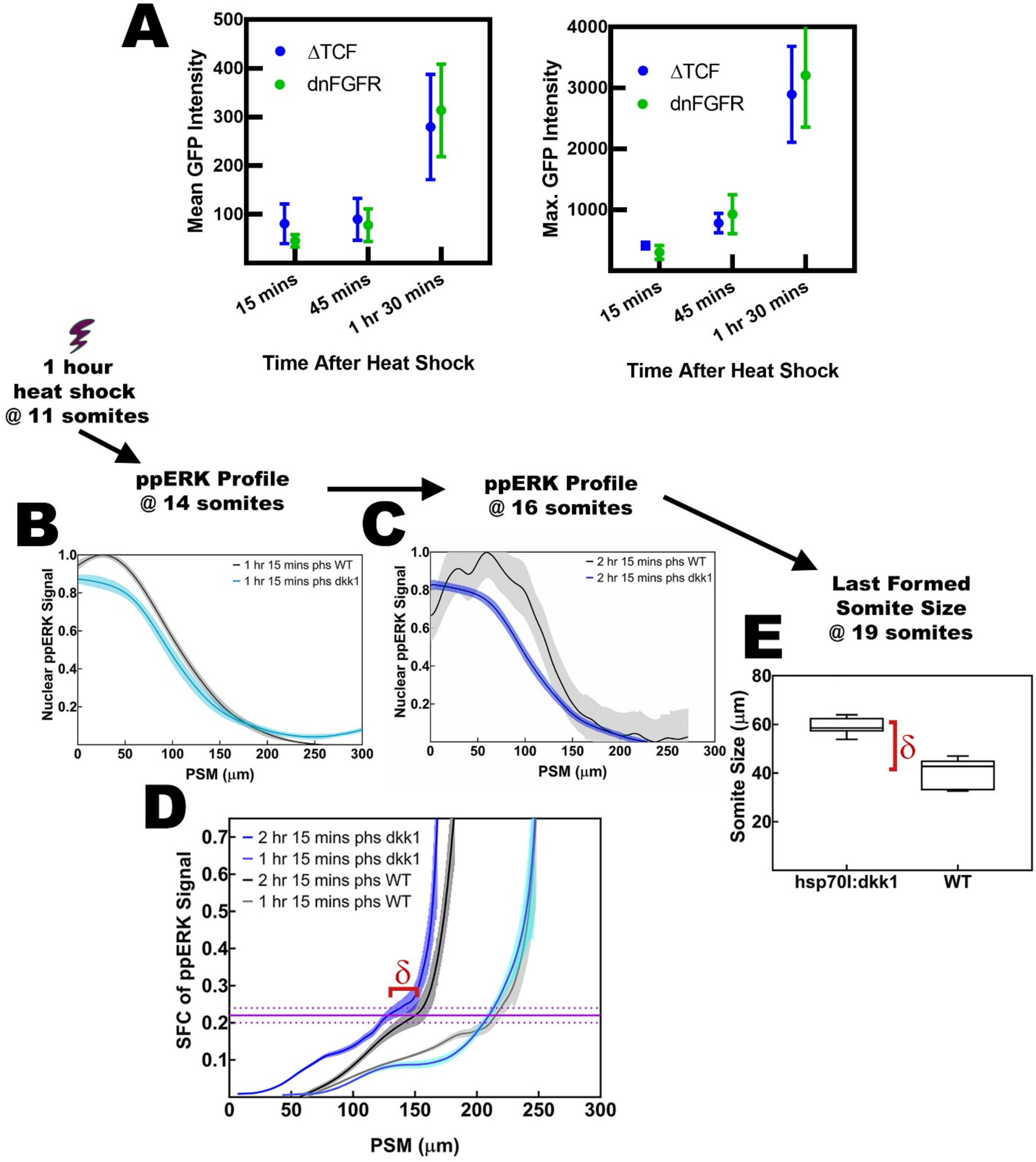

Figure S5. Wnt Signaling Influences Segmental Determination by Acting on the SFC of FGF Signaling, Related to Figure 5

(A) Mean and Maximum GFP intensity accumulated similarly in both *hsp70l:tcf7l1a-GFP* and *hsp70l: dnfgfr1a-EGFP* lines (n=3 and 3, respectively). Error bars indicate s.e.m. (B, C) Perturbation of Wnt signaling affect FGF SFC. Levels of ppERK (green) at 14 (B, n=28) or 16 (C, n=35) somite stages following 1 hour heat-shock (starting at 11 somite stage) in the *hsp70l:dkk1b-GFP* relative to control data (black) for same stages, ([B, n=14] and [C, n=10]). (C) Change of somite sizes following the heat-shock inhibition of Wnt signaling between 12 and 14 somite stages. (D) The SFC of FGF signaling shifted more posteriorly (δ=16+/-2 μm, dark blue) as compared to wild type (black) by 2.25 hours post heat-shock (inhibition of Wnt signaling), but not earlier (light colors), determining size of the first large somite to be formed three cycles later. (B-D) Shaded regions indicate s.e.m. Posterior is left. (E) The increase of somite size, at 19 somite stage, due to Wnt inhibition as quantified from nuclear staining pictures δ=17+/-3 μm (n=15) matches the shift of SFC of ppERK at 16 somite stage (D). Box plots show quartiles of data.

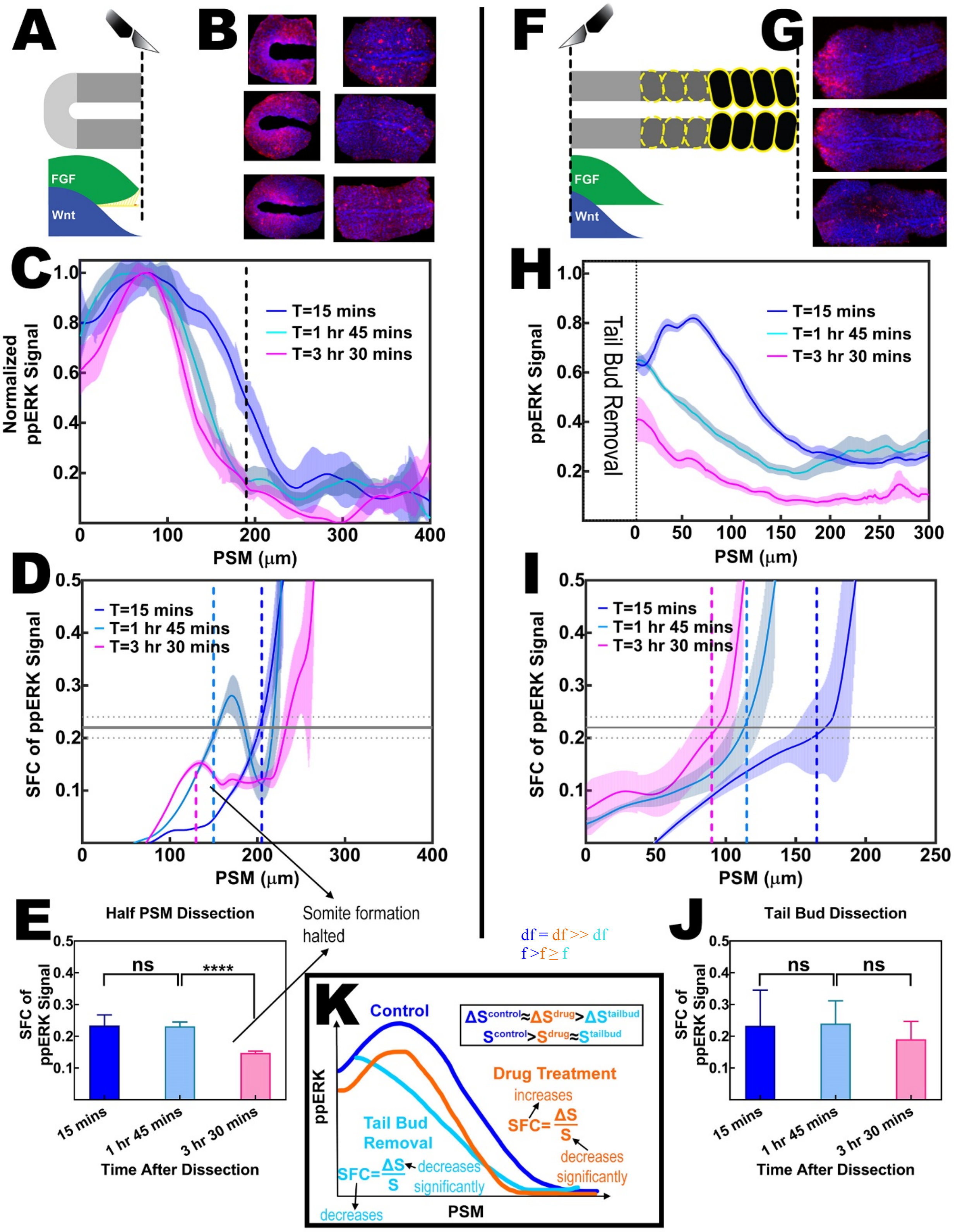

Figure S6. Spatial Fold Change of FGF Signaling Encodes Positional Information for Different Dissections of Explants, Related to Figures 2, and 6

(A, B) ppERK stainings (B) of half PSM explants and their matching anterior PSM halves. (C, D) Normalized levels (C) and the SFC (D) of ppERK quantified (n = 8, 8, and 8 at 15 min, 1.75 hours,and 3.5 hours post surgery). (A, C) Vertical dashed line is the surgery position. (D) Vertical dashed lines are the determination front positions as anticipated from determined somite sizes. (E) The SFC level of ppERK is conserved at the determination front position at 15 min, and 1.75 hours post-surgery when segments successfully formed. Strikingly, in spite of the fact that the PSM is not totally depleted and ppERK activity still establish a gradient, segmentation halts as the SFC of ppERK fails to pass a critical level at 3.5 hours post surgery. (F, G) ppERK stainings (G) of tailbud-truncated explants (F). (H, I) Measured levels (F) and the SFC (G) of ppERK activity (n = 6, 8, and 8 at 15 min, 1.75 hours, and 3.5 hours post surgery). (I) The SFC level of ppERK is conserved at the determination front position at 15 min, 1.75 hours, and 3.5 hours post surgery. Vertical dashed lines are the determination front positions as anticipated from determined somite sizes. Posterior end of the PSM are matched for all stages. Shaded regions and error bars indicate s.e.m. (K) Two different experiments decreasing FGF signaling result opposite effects on somite sizes. Tail bud removal decreases somite sizes whereas drug inhibition of FGF receptor increases. For a specific PSM position, SFC of FGF signaling increases in drug treatment whereas decreases in tail bud removed explants. Posterior is left.

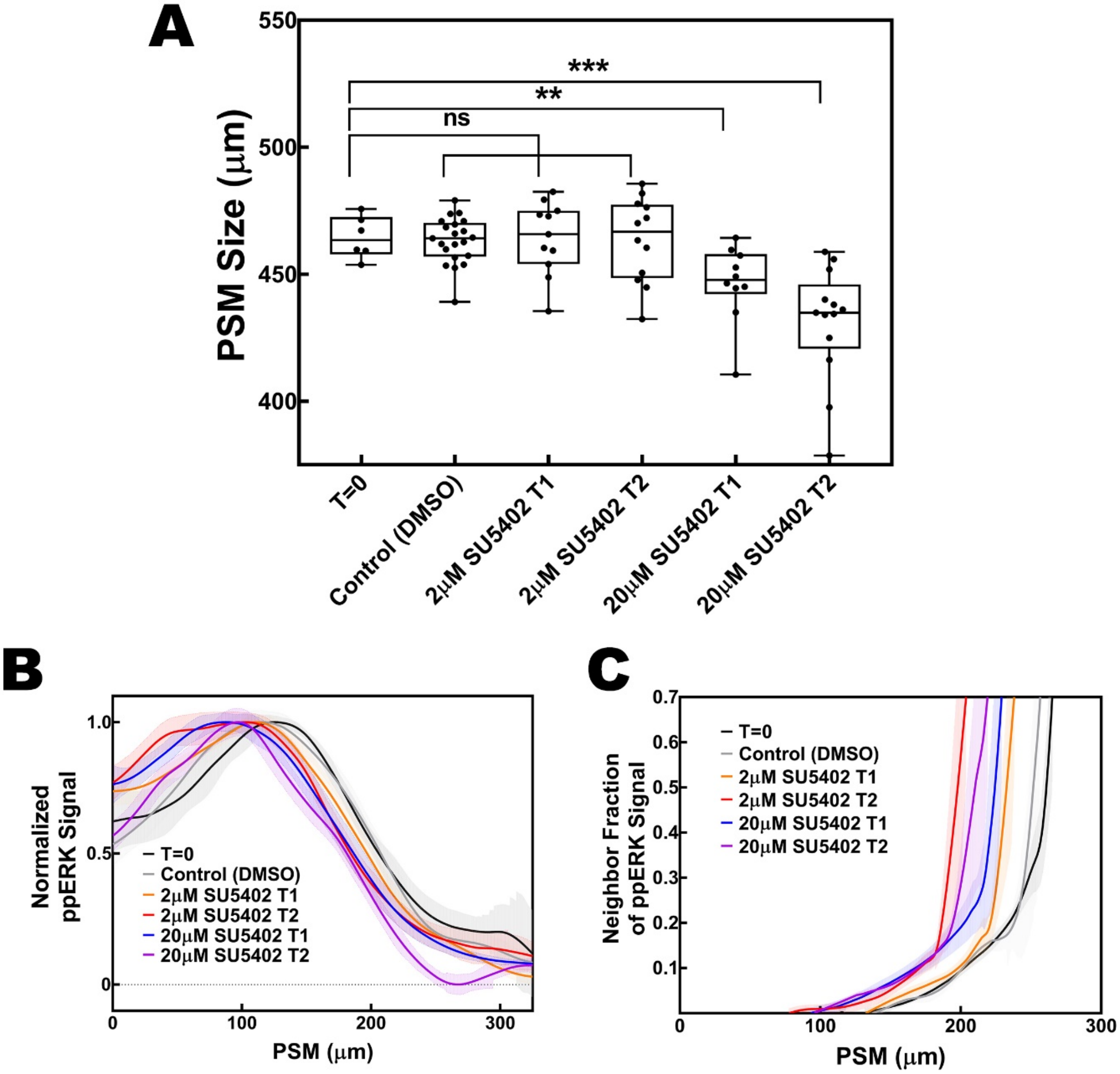

Figure S7. Pharmacological Blocking of FGF Signaling Acts in a Dose Dependent Manner, Related to Figure 6

Embryos were treated with either low (2 μM) or high (20 μM) dose SU5402, or with corresponding DMSO concentrations as control, continuously over 1 or 2 segmentation cycles (T1 and T2). Embryos at 12 somite stage, right after the treatments, were fixed for immunostaining. (A) Sizes of the PSM following low dose SU5402 (n=11) or DMSO (n=21) treatments did not differ from the untreated embryos fixed at same somite stage (T=0, n=6). High dose SU5402 (n=12) treatment resulted in gradually shorter PSM tissue (p=0.0085 and 0.0002, non-parametric student t-test) indicating reduction of axial elongation speed. (B) Changes in the FGF signaling are quantified with immunostaining of normalized nuclear ppERK levels. Inhibition of FGF signaling resulted posterior shift of normalized gradients. High dose inhibition resulted posterior shift immediately within the first segmentation cycle whereas low dose SU5402 caught the shift in two cycles. (C) The SFC of FGF signaling shifted gradually over two cycles for low dose treatment determining bigger somite in the second cycle of the treatment. The shift for the high dose treatment was similar for both short and high duration treatments showing somite sizes reduce back to normal after a single large somite, due to reduction of axial elongation speed. Shaded regions indicate s.e.m. Posterior is left.

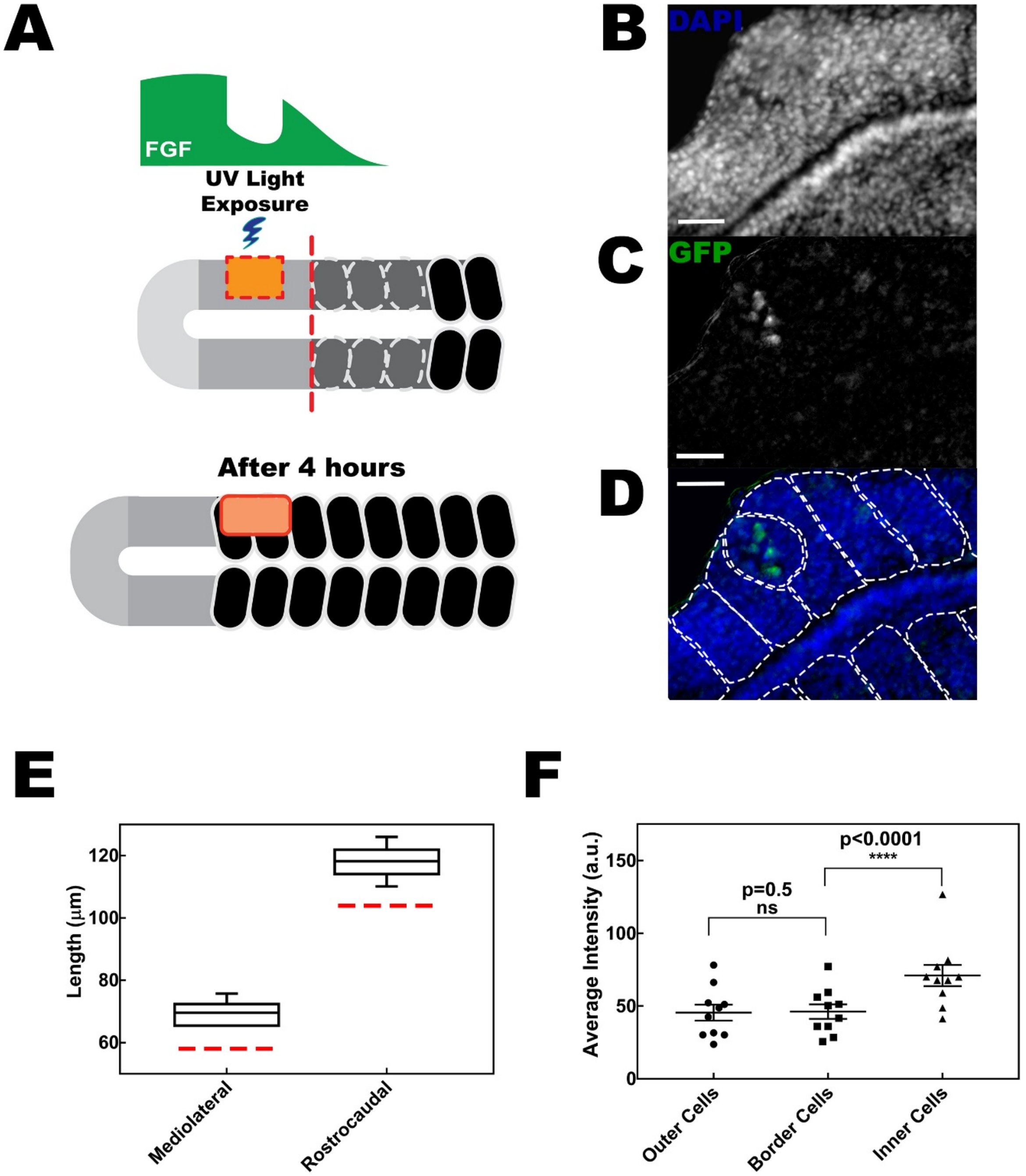

Figure S8. Local Perturbation of FGF Gradient Results Formation of Artificial Somites with Neighboring Cells, Related to Figure 7

(A) Sketch of local heat-shock experiments. UV light exposure of a window of cells (orange rectangle) in the undetermined posterior PSM results a well-like drop of FGF signal gradient (green) due to local heat-shock induced in the *hsp70l:dnfgfr1a-EGFP* transgenic line. Cells effected from the local heat-shock group together and form a precocious somite (bottom). (B-D) Representative nuclear (B) and heat-shock induced GFP (C) and overlaid (D) images of the mesoderm tissue. A circular precocious somite is formed (bottom, overlay) by cells effected from the heat-shock and their peripheral neighbors. (E) Measurement of precocious somite sizes for mediolateral (left, n=7) and rostrocaudal (right, n=14) alignments of rectangular pinhole used in the local heat-shock. Red dashed lines show the sizes of the pinhole measured directly through the microscope. (F) Maximum intensity projection of z-section GFP images (n=10) are quantified in three masks as cells outside the precocious somite (left, outer cells), cells at the internal periphery of the somite (middle, border cells) and cells inside the somite (right, inner cells). Signal from the internal border cells are not significantly different (paired t-test) from the background signal of outer cells. Error bars indicate s.d. Posterior is left.

Table S1

Parameter description	Symbol	Value / Range of values / Calculation
Setup parameters		
Lattice step size		1 cell
Simulation box (1-D)		85 cells
Total simulated time		18 hours (31 clock cycles)
Simulation time unit	stu	30 secs (70 stu=1 clock cycle)
Runge-Kutta time unit		0.3 secs (1 STU=100 iterations)
Size of tail bud		15 cells
Cell diameter		8 μm
Tail bud elongation speed	V_g	$1.143\ \mu m/min$ / variable array / zero for NG
Time delay parameters		
LIG translation time delay	trlatedel	7 mins
SIG activation time delay	actdel	7 mins
INH activation time delay	reprdel	14 mins
Held parameters		
mLIG transcription rate	k_{mLIG}^{syn}	$0.6\ s^{-1}$ (zeroth order)
LIG translation rate	k_{LIG}^{syn}	$0.06\ s^{-1}$ (first order)
SIG translation rate	k_{SIG}^{syn}	$1.2\ s^{-1}$ (zeroth order)
INH translation rate	k_{INH}^{syn}	$0.012\ s^{-1}$ (second order)
mLIG degradation rate	k_{mLIG}^{deg}	$0.0012\ s^{-1}$ (first order)
LIG degradation rate	k_{LIG}^{deg}	$0.012\ s^{-1}$ (first order)
COMP degradation rate	k_{COMP}^{deg}	$0.024\ s^{-1}$ (first order)
SIG degradation rate	k_{SIG}^{deg}	$0.012\ s^{-1}$ (first order)
INH degradation rate	k_{INH}^{deg}	$0.12\ s^{-1}$ (first order)
Variable parameters		
Ligand diffusion speed	D	$3 - 30\ \mu m/min$ (0.2 cell/stu step)
Ligand receptor binding rate	k_{LIG}^{bin}	$0.0012 - 0.012\ s^{-1}$ (second order)
SIG^- activation rate	k_{SIG}^{act}	$0.0018 - 0.018\ s^{-1}$ (second order)
SIG^+ deactivation rate	k_{SIG}^{deact}	$0.0018 - 0.018\ s^{-1}$ (second order)

Table S1. Description of Simulation Parameters, Related to Figure 3
